# InstaPrism: an R package for fast implementation of BayesPrism

**DOI:** 10.1093/bioinformatics/btae440

**Published:** 2024-07-05

**Authors:** Mengying Hu, Maria Chikina

**Affiliations:** Department of Computational and Systems Biology, University of Pittsburgh, Pittsburgh, 15260, United States; Joint Carnegie Mellon - University of Pittsburgh Computational Biology PhD Program, University of Pittsburgh, Pittsburgh, 15260, United States; Department of Computational and Systems Biology, University of Pittsburgh, Pittsburgh, 15260, United States; Joint Carnegie Mellon - University of Pittsburgh Computational Biology PhD Program, University of Pittsburgh, Pittsburgh, 15260, United States

## Abstract

**Summary:**

Computational cell-type deconvolution is an important analytic technique for modeling the compositional heterogeneity of bulk gene expression data. A conceptually new Bayesian approach to this problem, BayesPrism, has recently been proposed and has subsequently been shown to be superior in accuracy and robustness against model misspecifications by independent studies; however, given that BayesPrism relies on Gibbs sampling, it is orders of magnitude more computationally expensive than standard approaches. Here, we introduce the InstaPrism package which re-implements BayesPrism in a derandomized framework by replacing the time-consuming Gibbs sampling step with a fixed-point algorithm. We demonstrate that the new algorithm is effectively equivalent to BayesPrism while providing a considerable speed and memory advantage. Furthermore, the InstaPrism package is equipped with a precompiled, curated set of references tailored for a variety of cancer types, streamlining the deconvolution process.

**Availability and implementation:**

The package InstaPrism is freely available at: https://github.com/humengying0907/InstaPrism. The source code and evaluation pipeline used in this paper can be found at: https://github.com/humengying0907/InstaPrismSourceCode.

## 1 Introduction

Bulk tissue transcriptome represents a mixture of gene expression signals from heterogeneous cell populations. The process of “deconvolution” aims to computationally separate these mixture signals and provide estimates of cell-type abundance and, in some cases, gene expression profiles at cell-type resolution. This problem is particularly of interest in cancer profiling as tumor samples are composed of malignant and non-malignant cells (the tumor microenvironment or TME). Understanding the interplay between malignant cells and the TME is an active area of research with therapeutic potential (González-Navajas *et al.* 2021, Raskov *et al.* 2021).

Among numerous deconvolution methodologies that have been developed so far, BayesPrism (Chu *et al.* 2022) provides a novel Bayesian deconvolution framework, using scRNA-seq from similar tissue samples as priors. Comprehensive analysis both in the original BayesPrism paper (Chu *et al.* 2022) and independent benchmarking studies (Hippen *et al.* 2023, Tran *et al.* 2023, Hu and Chikina 2024) have demonstrated the superiority of BayesPrism for deconvolution. In particular the approach is highly robust against different sources of model misspecification, such as biological heterogeneity within cell-types (Hu and Chikina 2024) or technical differences between single cell reference and bulk (Hippen *et al.* 2023).

However, BayesPrism’s dependency on Gibbs sampling imposes a considerable constraint on its computational efficiency. For example, for a bulk expression matrix of 100 samples, BayesPrism needs hours of processing time, whereas competing methods take only seconds. Additionally, the memory demand for BayesPrism is substantial, given its need to accommodate the deconvoluted 3D cell-type-specific expression data. Together, these factors limit the application of BayesPrism to large scale studies.

To address these limitations, we present InstaPrism, an R package that provides fast implementation of BayesPrism. Maintaining the same conceptual framework and corresponding generative model, InstaPrism replaces the time-consuming Gibbs sampling steps with a fixed-point algorithm. It produces nearly identical deconvolution results while reducing the running time to seconds. Moreover, InstaPrism optimizes memory utilization by compressing the 3D cell-type-specific information into a 2D scaling matrix, which remarkably decreases memory demands. The package also includes a set of built-in references generated from extensive processing of large single-cell studies, providing ready-to-use options for deconvolution analysis across a variety cancer types. Lastly, we offer a general guideline for reference construction and an evaluation pipeline to streamline performance assessment, facilitating deconvolution analysis in practice.

## 2 Materials and methods

InstaPrism follows the same conceptual framework as BayesPrism, which views the deconvolution problem as an instance of topic modeling. In this context, individual reads in bulk RNA-seq are analogous to words, each bulk RNA-seq sample corresponds to a document, each cell state is a topic, and each gene is an element of the vocabulary. Here, the innovative idea of “cell state,” which was originally proposed by BayesPrism, aims to reflect various gene expression patterns within a single cell type, instead of representing a cell type with a uniform profile. The posteriors of different cell states are then summed up to represent the corresponding cell type-level posteriors. In Supplementary Text S1, we provided a detailed description of the InstaPrism model.

### 2.1 Input

The InstaPrism model requires two input data for the deconvolution task:

- The bulk expression data $X_{G\times N}$ (a gene by sample matrix) for deconvoltion analysis. This data should be in non-log transformed scale and users need to have preliminary information about the tissue source, such as whether it is from peripheral blood mononuclear cell (PBMC) samples or breast cancer tumors.
- A reference profile containing prior knowledge from the same tissue type. Specifically, this profile includes a reference matrix $A_{G\times S}$ indicating the probability of a gene being expressed in each cell state, with $\sum_{g}A_{g,s}=1$ for all cell state s∈[1,S], and mapping information that indicates which cell type each cell state corresponds to. The posterior estimates for cell states within the same cell type will later be aggregated to yield the final posterior estimates at the cell type level.

The reference profile can be generated *de novo* using the *refPrepare()* function from InstaPrism, with user-provided scRNA-seq data. Specifically, this function requires raw scRNA-seq expression data (in non-log transformed scale) along with cell labels at both cell type and cell state levels as input. The *refPrepare()* function then calculates the $A_{G\times S}$ matrix by averaging gene expression profiles of the same cell state (using the maximum likelihood estimates) and provides the mapping information that links cell states to their corresponding cell types.

Note that this *de novo* reference construction step involves multiple hyperparameter choices, including the selection of the scRNA-seq dataset, specifications for cell types and cell states, and subclustering hyperparameters when cell state information is absent from the source scRNA-seq. In Supplementary Text S2-S3, we discussed how different hyperparameter choices affect model performance and provided general guidelines on reference construction.

To streamline the reference construction step, we have provided a set of built-in references (Table 1, Supplementary Tables S1 and S2) tailored for a wide range of cancer types. The references, curated through the processing of 2 133 334 single cells from seven large single-cell studies, capture an extensive array of cell-type and cell-state expression profiles and reflect the complexity of the tumor microenvironment. The built-in references are now accessible with the *InstaPrism_reference()* function.

**Table 1.** Built-in references in InstaPrism.

Reference name	Tumor type	Number of cells for reference construction	Number of cell types/cell states	Source
BRCA_refPhi	Breast cancer	100 064	8/76	Wu *et al.* (2021)
CRC_refPhi	Colorectal cancer	371 223	15/98	Pelka *et al.* (2021)
GBM_refPhi	Glioblastoma	338 564	10/57	Ruiz-Moreno *et al.* (2022)
LUAD_refPhi	Lung adenocarcinomas	118 293	13/77	Xing *et al.* (2021)
OV_refPhi	Ovarian cancer	929 690	9/40	Vázquez-García *et al.* (2022)
RCC_refPhi	Renal clear cell carcinoma	270 855	11/106	Li *et al.* (2022)
SKCM_refPhi	Skin Cutaneous Melanoma	4645	8/23	Tirosh *et al.* (2016)

### 2.2 Usage and output

Upon preparation of the necessary inputs, the deconvolution task can be accomplished with the *InstaPrism()* function. Specifically, this function returns the posterior fraction estimates at both the cell state and cell type levels. Note that this function does not directly output the posterior estimates of cell type-specific expression, mainly due to memory efficiency considerations. Instead, it returns a 2D scaling matrix with compressed information which can later be utilized to reconstruct the cell type-specific information.

Depending on users’ interests, the cell type-specific expression can be derived using the *reconstruct_Z_ct_initial()* function, which returns a gene by sample matrix specifying the cell-type specific expression for a cell type of interest, or through the *get_Z_array()* function, which outputs the fully deconvolved 3D cell-type specific expression $Z_{G\times N\times K}$ (a sample by gene by cell type array).

As a supplementary feature, InstaPrism incorporates the capability of reference updates with the *InstaPrism_update()* function, matching the default deconvolution option in BayesPrism. This process involves constructing an updated reference matrix using the posterior estimates $Z_{G\times N\times K}$ from the first-round deconvolution results and updating the fraction estimates accordingly. However, our empirical testing results indicate that utilizing an updated reference does not necessarily enhance deconvolution accuracy (Supplementary Text S1); hence, we have designated this module as optional.

### 2.3 The InstaPrism algorithm

Adhering to the same conceptual framework established by BayesPrism (Chu *et al.* 2022), InstaPrism also solves the deconvolution problem as a topic model analogy. InstaPrism differs from BayesPrism in how topic modeling is performed. Specially, for one optimization step in topic modeling, BayesPrism requires an additional iterative sampling process that loops through every gene individually to perform the sampling; in contrast, InstaPrism simplifies this process by utilizing a single vectorized calculation that directly computes only the mean of the corresponding distribution, which greatly reduces the computation load.

In other words, the InstaPrism method can be seen as a derandomization of the original BayesPrism method. It is methodologically similar to BayesPrism but eliminates the sampling step, thereby maintaining equivalent deconvolution results. A detailed side-by-side comparison of two methods is available at Supplementary Text S1.

### 2.4 Reference evaluation pipeline

We note that the reference profile is an essential input to the model and also a hyperparameter that impacts model performance. In practice, the construction of reference profiles involves multiple choices, including the selection of scRNA-seq data, cell-type/cell-state identification and other classification hyperparameters such as the granularity of cell states and clustering parameters. In Supplementary Text S2 we provided practical guidance on these issues and introduced a reference evaluation pipeline. This pipeline efficiently tests reference performance using both simulated and real bulk data, facilitating comparisons across multiple references and aiding in the optimization of reference construction and hyperparameter selections.

### 2.5 Comparison with BayesPrism

**Figure 1.**
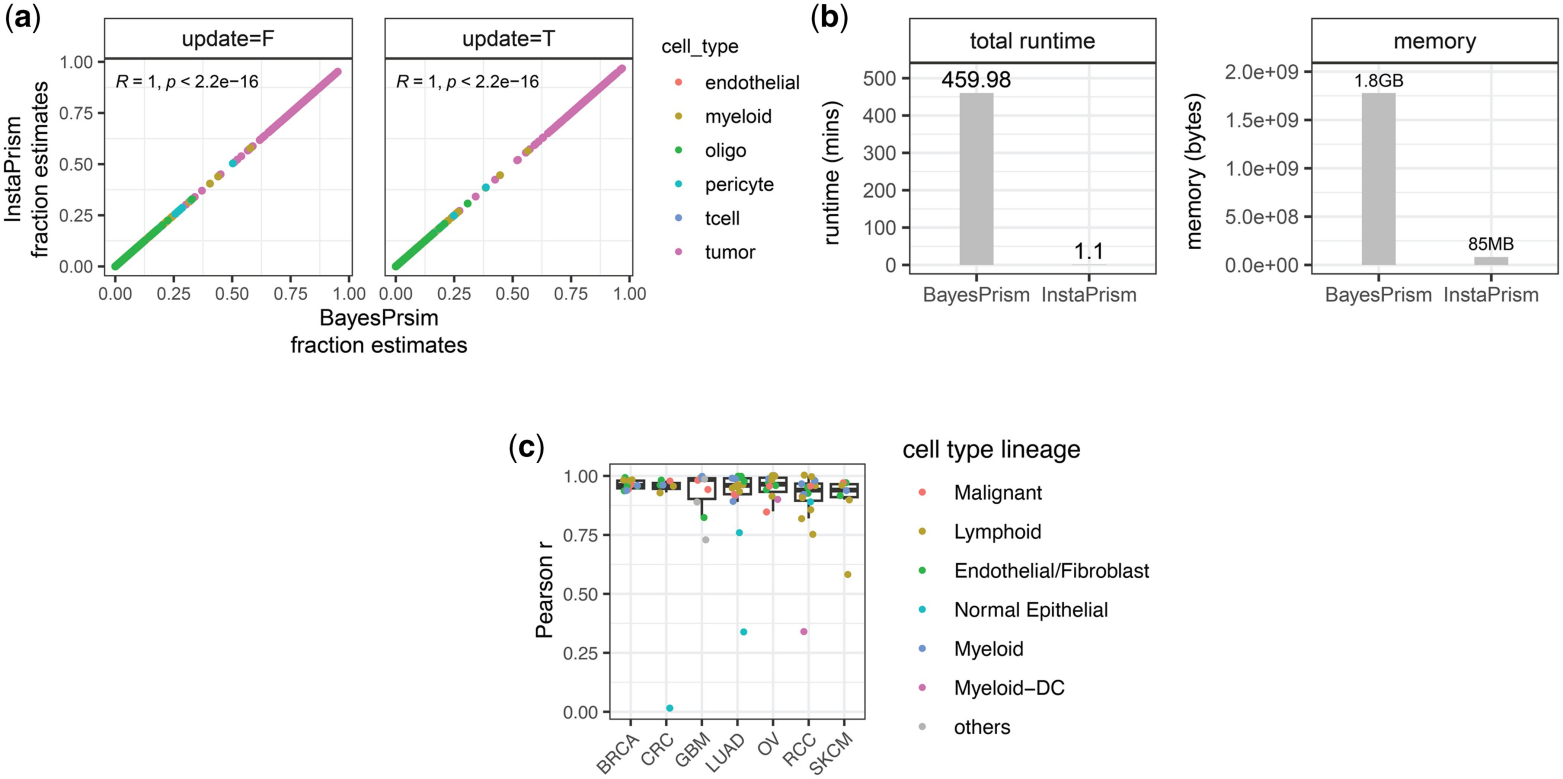
Performance of InstaPrism. (a) Scatter plot comparing the cell type fraction estimates between BayesPrism and InstaPrism under two reference update modes, with each dot represents a fraction estimate and colored by cell type. (b) Computational efficiency comparison. (c) Box plot showing the performance of InstaPrism built-in references, as evaluated by Pearson correlation between the estimated and ground truth cell type fractions in simulated datasets. Each dot corresponds to a cell type in the reference. Cell types belonging to the same lineage are depicted in the same color. In (a) and (b), results are based on deconvolution analysis using the tutorial data provided by BayesPrism, conducted with the default parameter settings. In (c), results are based empirical testing from simulated bulk datasets.

Using the tutorial data provided in the original BayesPrism package (https://github.com/danko-lab/bayesprism/tree/main/tutorial.dat), which consists of both bulk data for deconvolution and scRNA-seq data for reference construction, we performed deconvolution analysis using both InstaPrism and BayesPrism. This analysis involved deconvolving 169 bulk samples from the TCGA-GBM cohort, which are presented in raw count format. Our results showed that both the cell-type fraction estimates (Fig. 1a) and the 3D cell type-specific expression estimates (data not shown) produced by the two methods are nearly identical. Additionally, a trace plot comparing model update details (Supplementary Fig. S1) revealed that both methods exhibit nearly identical update trajectories over iterations for fraction estimates, which further confirmed that InstaPrism is equivalent to BayesPrism minus the sampling methodologically.

While the deconvolution results are nearly identical, InstaPrism significantly enhances computational efficiency, reducing the deconvolution runtime from hours to minutes and decreasing memory usage from gigabytes to megabytes (Fig. 1b), thereby demonstrating its superior computational efficiency without compromising accuracy.

### 2.6 Performance of the built-in references

We extensively tested the performance of the precompiled references by applying them to simulated bulk datasets (Supplementary Text S5). For each built-in reference, the validation datasets were simulated using scRNA-seq data from the same tumor type but distinct from those used in reference construction. This approach is crucial as it ensures no information flow between reference construction and bulk simulation, replicating real-world deconvolution scenarios when there are inevitable technical differences between the reference data and the bulk samples being analyzed (Garmire *et al.* 2024).

In Fig. 1c, we summarized the performance of the references, evaluated by the Pearson correlation between the estimated and ground truth cell type fractions. Across the validation datasets, the estimated fractions closely align with the ground truth fractions, achieving an average Pearson correlation of 0.92 per cell type. Despite some exceptions with rare cell types, each reference generally performs well and shows good generalizability.

Detailed descriptions of the validation datasets and additional results from applying the built-in reference to real TCGA tumor data are available at Supplementary Text S5.

## 3 Conclusion

InstaPrism provides a fast implementation of the Bayesian-based deconvolution model. It takes advantage of the conceptual Bayesian framework proposed in BayesPrism but reformulates it as closed-form updates. The deconvolution results of InstaPrism is highly comparable with BayesPrism and can be implemented for large-scale deconvolution study. The improved running time will also enable exploration of how different cell-state and reference specifications affect the results. Moreover, InstaPrism’s built-in references, which thoroughly summarize recent large scRNA sequencing work, capture a broad spectrum of cell-type and cell-state specific insights and provide a ready-to-use resource for analyzing bulk data across a variety of cancer types. Lastly, the general guidelines for reference construction and the accompanying evaluation pipeline that we provide are designed to enhance real-world deconvolution practices, enabling researchers to efficiently apply InstaPrism in diverse scientific environments.

## Supplementary Material

btae440_Supplementary_Data

## Data Availability

The InstaPrism R package is freely available at: https://github.com/humengying0907/InstaPrism. The source code and evaluation pipeline used in this paper are available at: https://github.com/humengying0907/InstaPrismSourceCode. The dataset used for performance comparison underlying this article is available at https://github.com/danko- lab/bayesprism/tree/main/tutorial.dat.orMore information on the data sources are stated in the main text above.
