# Supplementary material for: InstaPrism: an R package for fast implementation of BayesPrism
Source: Bioinformatics. 2024 Jul 5;40(7):btae440. doi: 10.1093/bioinformatics/btae440 (PMC11245312; doi:10.1093/bioinformatics/btae440)
Supplement: btae440_Supplementary_Data [file btae440_supplementary_data.pdf]

Supplementary Information

InstaPrism: an R package for fast implementation of BayesPrism

Mengying Hu[1,2], Maria Chikina[1,2,*]
[1]Department of Computational and Systems Biology, University of Pittsburgh School of Medicine, Pittsburgh, PA, USA, [2]Joint CMU-Pitt PhD Program in Computational Biology, Pittsburgh, PA, USA
* Corresponding author. mchikina@gmail.com

Contents

Supplementary Texts **2**
Text S1: InstaPrism vs. BayesPrism from a methodological perspective 2
Text S2: Practical guidance on reference construction 6
S2.1. Collect scRNA data from matched tissue type 6
S2.2. Cell state specification 7
S2.3. Reference evaluation pipeline 7
S2.4. Recommendations on reference selection 9
Text S3: Impact of cell state specification on model performance 9
S3.1. Impact of duplicated reference 9
S3.2. Impact of cell state granularity 10
S3.3. Impact of missing cell types 11
S3.4. Selection of cell types in the reference 13
Text S4: Model convergence 14
Text S5: Construction & Validation of InstaPrism Built-in reference 16
S5.1. Validation using simulated data 16
S5.2. Validation using TCGA data 18

Supplementary Tables **21**
Table S1: scRNA data used for reference construction and bulk simulation 21
Table S2: Overview of cell type and cell state information in built-in reference 22
Table S3: Summary of TCGA datasets used in reference evaluation 28

References **29**

Supplementary Texts

Text S1: InstaPrism vs. BayesPrism from a methodological perspective

Adhering to the same conceptual framework established by BayesPrism [1], InstaPrism also interprets the deconvolution problem as a topic model analogy, with reads in bulk RNA-seq equivalent to a word, each bulk RNA-seq sample equivalent to a document, each cell state equivalent to a topic and each gene equivalent to a vocabulary. The topic modeling is performed at the cell state level to accommodates multiple gene expression subtypes (referred to as "cell states") within a cell type, instead of representing a cell type with a uniform expression profile. The model directly outputs posterior estimates for fractions (document-topic distribution) and gene expression (document-vocabulary distribution) at cell state levels, which are then summed up to yield the final posteriors at the cell type levels.

When using topic modeling for deconvoltuion, both methods maintain the following simplifications compared to traditional latent Dirichlet allocation (LDA) process [2]: **1)** The topic-word distribution is predefined and remains constant throughout the model. This eliminates the need for further inference of this parameter [3], reducing computational costs and allowing for independent inference for each "document" (bulk sample); **2)** The Dirichlet prior α of the LDA is non-informative, making the posterior mainly driven by the likelihood. BayesPrism adopts a fixed and non-informative Dirichlet prior ($\alpha = 10^{-8}$) in their model, imposing negligible effect when sampling the document-topic distribution (the cell state fractions $\theta_{S\times1}$ in this context) from the Dirichlet distribution. In InstaPrism, we adhere to the same idea of a non-informative Dirichlet prior and directly update θ without information from the Dirichlet prior.

InstaPrism differs from BayesPrism in how topic modeling is performed. While BayesPrism relies on Gibbs sampling in model optimization, InstaPrism directly computes the expected mean without the need for sampling.

To illustrate how InstaPrism algorithm differs from BayesPrism algorithm, we consider a single bulk gene expression sample with G genes, $X_{G\times1}$, and a cell-state reference matrix, $A_{G\times S}$, where S is the total number of cell-states. BayesPrism infers two variables, the proportions of cell-states in X, $\theta_{S\times1}$ and cell-state specific expression $Z_{G\times S}$. In the InstaPrism algorithm we keep track of another matrix called $B_{G\times S}$ which depends on both the static reference input A and the current proportion estimate θ. Each row of B is the mean of a multinomial distribution for assigning the reads of gene $g \in [1, G]$ to the S different cell-states. This is a reformulation of the two-step multinomial model described in BayesPrism. In particular, at each iteration we maintain $\sum_s B_{g,s} = 1$ for all $g \in [1, G]$ by normalizing the rows.

Overall the parameters of InstaPrism/BayesPrism can be summarized as follows:

- Input: $X_{G\times1}$: gene expression in a given bulk sample
- Input: $A_{G\times S}$: gene expression reference matrix, with $\sum_g A_{g,s} = 1$ for all cell state $s \in [1, S]$
- Intermediate variable (for InstaPrism only): $B_{G\times S}$: probability of assigning a read in gene $g \in [1, G]$ to a cell-state $s \in [1, S]$
- Gibbs sampling parameter (for BayesPrism only): $\alpha = 10^{-8}$, a non-informative Dirichlet prior
- Output: $\theta_{S\times1}$: cell-state fractions
- Output (for InstaPrism only): $W_{G\times1}$: scaling matrix to reconstruct $Z_{G\times S}$
- Output (for BayesPrism only): $Z_{G\times S}$: cell-state specific gene expression

We use notation $Z_{g,}$ and $Z_{,s}$ to indicate genes/rows and cell-states/columns of Z respectively. Given these variable definitions, we present a detailed comparison of two algorithms as follows:

Algorithm 1 InstaPrism: Fixed-point algorithm

Require: $A \in \mathbb{R}^{G\times S}$, $X \in \mathbb{R}^{G\times 1}$
- $\theta_n \leftarrow 1/S$ ▷ initialize proportions
- $A'_{g,} \leftarrow A_{g,} \circ (1/\sum_s A_{g,s})$ ▷ normalize genes
- **while** $i < num.iter$ **do**
  - $B_{g,} \leftarrow A'_{g,} \circ \theta$ ▷ scale each cell reference by θ
  - $B_{g,} \leftarrow B_{g,} \circ (1/\sum_s B_{g,})$ ▷ normalize B as probabilities
  - $Z_{g,} \leftarrow X_g B_{g,}$ ▷ distribute reads for gene
  - $\theta_{new} \leftarrow \sum_g Z_{,s}$ ▷ total reads assigned to cell-state s
  - $\theta_{new} \leftarrow \theta_{new}/\sum(\theta_{new})$ ▷ normalize to get proportions
  - $\theta \leftarrow \theta_{new}$
  - $i \leftarrow i+1$
- **end while**
- $W = A' \times \theta \times Diag(X)^{-1}$ ▷ derive the scaling matrix
- **return** $\theta_{S\times 1}, W_{G\times 1}$

Algorithm 2 BayesPrism: Gibbs Sampling

Require: $A \in \mathbb{R}^{G\times S}$, $X \in \mathbb{R}^{G\times 1}$
- $\theta_n \leftarrow 1/S$ ▷ initialize proportions
- **while** $i < num.iter$ **do**
  - $B_{g,} \leftarrow A_{g,} \circ \theta$ ▷ initialize reads probability
  - **for** $g \leftarrow 1$ to G **do**
    - $Z_{g,} =$ Multinomial$(n = 1, size = X_g, prob = B_g)$ ▷ distribute reads by sampling
  - **end for**
  - $\theta_{new} \leftarrow$ Dirichlet$(\sum_g Z_{,s} + \alpha)$ ▷ update θ by sampling
  - $\theta \leftarrow \theta_{new}$
  - $i \leftarrow i+1$
- **end while**
- **return** $\theta_{S\times 1}, Z_{G\times S}$

The InstaPrism algorithm essentially finds θ, which is the fixed point of the above iterative updates. The fixed point θ has the property that it assigns each gene's read counts (or expression values) according to the renormalized $A'_{g,} \circ \theta$ and forms the deconvolved matrix $Z_{G\times S}$ that obeys the following equality:

$$\sum_{s=1}^{S} Z_{g,s} = X_g \quad \text{for each } g,$$

$$\frac{\sum_{g=1}^{G} Z_{g,s}}{\sum_{s=1}^{S}\sum_{g=1}^{G} Z_{g,s}} = \theta_s \quad \text{for each } s$$

The algorithm can also be seen as a derandomization of the original BayesPrism method by replacing the iterative sampling process for each gene, into a single vectorized computation. This means that instead of looping over all the genes to acquire the necessary input for θ update, the algorithm now achieves this with one single step. Despite this simplification, InstaPrism maintains performance integrity: both algorithms exhibit comparable fraction update trajectories (Figure S1), suggesting that InstaPrism is methodologically equivalent to BayesPrism minus sampling.

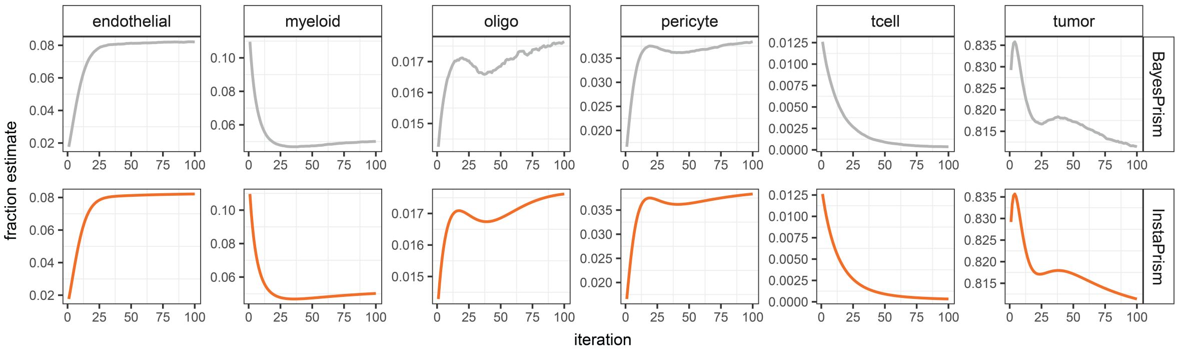

Figure S1: **Fraction update trajectory**. Lineplot showing how cell-type fractions change over iterations in BayesPrism and InstarPrism for one bulk sample from the tutorial data [4] provided in BayesPrism.

In InstaPrism, the cell state specific expression $Z_{G\times S}$ can be reconstructed by:

$$Z_{G\times S} = Diag(W_{G\times 1}) \times A'_{G\times S} \times Diag(\theta_{S\times 1}) \tag{1}$$

With the posterior estimates of fractions $\theta_{S\times 1}$ and gene expression $Z_{G\times S}$ at cell state level, both methods now calculate the final deconvolution estimates at cell type level as follows:

for each cell type k,

$$\theta_k = \sum_{s\in k} \theta_s \tag{2}$$

$$Z_{gk} = \sum_{s\in k} Z_{gs}, \tag{3}$$

We note that the comparison mentioned above mainly focuses on the algorithmic aspect. In real practice, another notable difference is the inclusion of the "reference update" step in the deconvolution process. While BayesPrism by default performs two rounds of topic modeling: initially with an scRNA-based reference and subsequently with an updated reference, InstaPrism provides deconvolution results using only the scRNA-based reference by default.

The "reference update" feature introduced by BayesPrism aims to utilize the shared information across bulk samples to refine cell type fraction estimates [1]. This process begins by using posterior estimates from the first round of deconvolution, specifically the cell-type specific expression $Z_{G\times N\times K}$ (the combined $Z_{G\times K}$ across N samples), to establish a new sample-specific reference profile ψ. The updated reference differs from single-cell based reference in the following ways:

1. The reference of malignant cells, denoted as $\psi_{mal_{g\times n}}$, is unique for each individual bulk sample. Specifically, for the gth gene of the nth sample,

$$\psi_{mal_{gn}} = \frac{Z_{gnt}}{\sum_g Z_{gnt}}, \text{ where } t \text{ indicate the malignant cell type}$$

2. The reference of the non-malignant cells (or environmental cells), denoted as $\psi_{env_{g\times k}}$, is shared across all bulk samples. Again, the calculation of ψ_{env} directly utilizes the deconvolved cell type-specific expression $Z_{G\times N\times K}$. Specifically, for the gth gene for the kth cell type, InstaPrism updates the reference as follows:

$$\psi_{\text{env}_{gk}} = \frac{\sum_{n=1}^{N} Z_{gnk}}{\sum_{g=1}^{G}\sum_{n=1}^{N} Z_{gnk}}$$

3. The updated reference no longer preserves information at the cell state level; instead, it only contains information at cell type level.

This updated reference is then subjected to topic modeling again to yield the updated posterior estimates. In InstaPrism, this functionality is available as an optional feature through the *InstaPrism_update()* function. This function provides updated posterior fraction estimates that are nearly identical to those from BayesPrism (data not shown), and additionally updates the posterior of gene expression, a feature not available in BayesPrism.

Given that the "reference update" step involves pooling information from input bulk samples, the quality of the updated reference can be highly input-dependent and there is yet no definite

answer to decide under what circumstances reference update is appropriate. It can be anticipated that the quality of the updated reference can be easily influenced by factors such as number of samples and the quality of the input.

Below, we present empirical testing results we obtained when comparing the deconvolution performance using initial reference (the InstaPrism built-in reference) and the updated reference, using a set of validation datasets that include both real bulk samples from the TCGA-cohorts and simulated samples (for validation datasets construction details, see Text S5; for datasets details, refer to Table S1 and S3). Although improvements were observed in tumor purity estimation [5, 6, 7] for the TCGA cohorts using the updated reference (Fig. S2a), the overall benefit remains uncertain since we don't have access to ground truth fractions for other cell types. It is likely that we achieve improved predictions for the malignant population only, but compromise predictions for other cell types. Additionally, it is likely that a large number of input samples (see Table S3 for details) is a key factor that leads to benefits from the updated reference, which is not typical in real-world deconvolution settings where input sizes are often limited.

In a separate evaluation (Fig. S2b), we tested the effect of reference updates using 12 simulated datasets (Table S1), each comprising 50 simulated samples with available ground truth fractions for all cell types. Indeed, in some datasets, such as the two simulated LUAD datasets, we observed that the reference update benefits predictions for malignant cells only, while decreasing prediction accuracy for other cell types. For 9 out of the 12 datasets, the updated reference worsens the overall estimation of the cell-type fractions, whereas in the remaining three datasets, it only slightly improves the estimations. Overall, no clear advantage of using a reference update is observed.

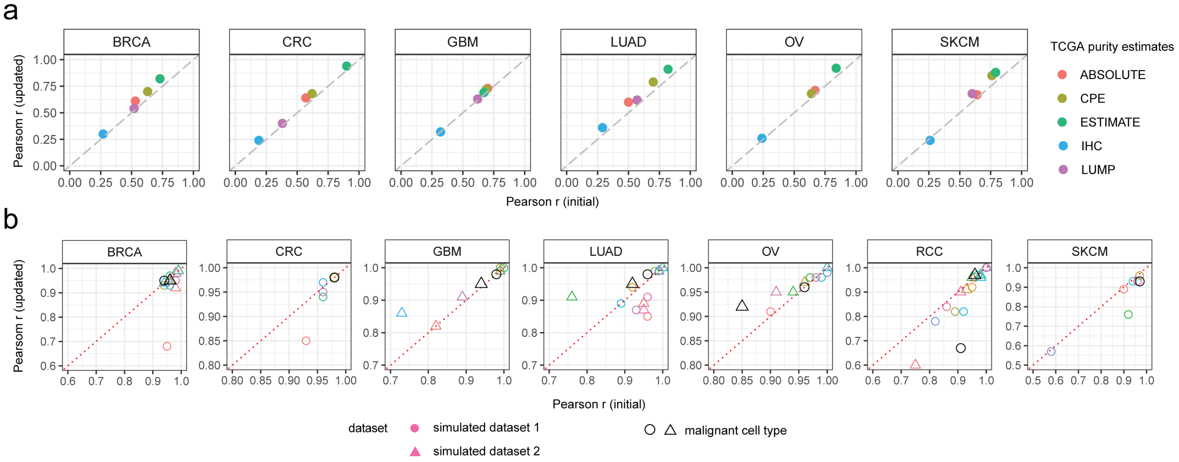

Figure S2: **Effect of reference update in validation datasets.** Scatter plot comparing the deconvolution performance, as evaluated by Pearson correlation, between scRNA-based reference (InstaPrism built-in reference) and updated reference. **a** Using real bulk samples from the TCGA cohorts, each dot represents the Pearson correlation between the estimated malignant fractions and one kind of TCGA tumor purity estimates, which serves as a proxy for ground truth malignant fractions. **b** Using simulated samples with known cell type fractions, each dot represents the Pearson correlation between the estimated and ground truth cell type fractions. Different colors correspond to different cell types, with the black color represents the malignant cell type specifically. Higher correlations indicate better deconvolution performance.

We also attempted to pool information at the cell state level to maintain the same granularity level of the scRNA-based reference (using cell-state specific expression $Z_{G\times N\times S}$ for ψ calculation); however, this approach proved even worse (data not shown). Taken together, given the input-dependent nature of "reference update" and our empirical test results, we recommend adhering to the scRNA-based reference for optimal performance, and suggest only considering updated reference for malignant estimation.

Text S2: Practical guidance on reference construction

While independent studies have demonstrated that the Bayesian deconvolution framework of BayesPrism/InstaPrism excels in accuracy and robustness for deconvolution tasks [8, 9], it's important to note that the model intrinsically requires a reference hyperparameter, denoted as $A_{G\times S}$, that specifies the event probability of the gth gene in sth cell state.

In InstaPrism, we provided a *refPrepare()* function to construct such reference using user-provided scRNA-seq data and cell annotation information. The function calculates the average expression values of single cells within the same cell states and then renormalizes these values such that the sum across genes is 1, yielding the final $A_{G\times S}$ reference matrix. This hyperparameter $A_{G\times S}$ encapsulates extensive prior knowledge on gene covariance structures and requires no subsetting of the marker genes. The proper configuration of this hyperparameter is important for model performance. Here we provide several guidelines for configuring this hyperparameter.

1. Collect scRNA data from matched tissue type

To begin with, one need to collect scRNA-seq data from matched tissue type, following the general assumption that the constructed reference will capture the major cell types present in the bulk sample [10]. The ideal scRNA-seq datasets should be of high quality, presented on a linear (non-log) scale, include pre-defined cell type annotations, and ideally contain sample-level annotations that specify the biological origin of the single cells. Available scRNA-seq databases for this purpose include, but are not limited to, the 3CA atlas [11], CellxGene [12], and the Single Cell Portal [13].

Specifically, opting for larger and higher quality datasets helps mitigate the dropout issue [14] commonly encountered in single-cell data. Given that reference construction relies on aggregating gene expression profiles across individual cells, a larger sample size can reduce the impact of dropout. The well-labeled single-cell datasets offer the advantage of efficiently identifying cell types and more detailed cell subtype (referred to as "cell state") information, which is crucial for effective reference construction. And the sample-level annotation information can aid in further finer-grained cell subclustering when needed.

We acknowledge that with recent advancement in single cell technology, the availability of scRNA-seq datasets has significantly increased. When multiple candidate datasets are available, one can utilize the evaluation pipeline we proposed (`https://github.com/humengying0907/InstaPrismSourceCode`) to systematically compare between references and select the one that is optimal.

2. Cell state specification

Cell state labeling information is another key component in $A_{G\times S}$ construction. It implies two layers of information: 1) the cell state of the single cell, and 2) the cell type to which this cell state is affiliated. This information is passed to the *refPrepare()* function to indicate which single cells to aggregate together to construct the $A_{G\times S}$ reference matrix, and is subsequently used to aggregate the posterior estimates of cell states within the same cell type, yielding the final posterior estimates at cell type level.

In practice, cell state information can be obtained from original single-cell annotations when subclustering information of the identified cell types is provided. When such information is missing and only cell type annotations are available, we recommend the users to use the *get_subcluster()* function provided in InstaPrism to get the fine-grained cell state information within each cell type. This function incorporates two subclustering methods: the "quickCluster" method from scran package [15] and the "SCISSORS" method [16]. Specifically, the "quickCluster" method offers a significant speed benefit for clustering, and the "SCISSORS" method includes a *SilhouetteScores.cutoff* parameter to determine cell types requiring re-clustering and can automatically determine optimal parameters for subclustering [16], providing a convenient and efficient way to capture the inherent subclustering structures within cell types. Additionally, for heterogeneous populations like malignant cells, one can assign cell state labels based on their source of origin (using patient identifiers), when other subclustering methods are computationally intensive. We recommend having at least 20 cells to represent each cell state, in line with the recommendations from BayesPrism.

We acknowledge that cell-type subclustering is an unsupervised problem (check next section for details), entailing various methodologies choices including granularity levels, clustering techniques and clustering hyperparameters. While there is no definite answer to this selection, the quality of resulting reference $A_{G\times S}$ can be assessed using the evaluation pipeline we provide, which helps determine the most appropriate methods to use.

3. Reference evaluation pipeline

To assess the performance of the reference, one can utilize the reference evaluation pipeline we provide, which includes the following steps:

1). Simulation of bulk samples with known proportions. Bulk samples with known cell type proportions are crucial for evaluating deconvolution performance, as they provide ground truth proportions for results comparison [10]. One common method to generate this type of data is to simulate bulk data by aggregating single cells in predefined proportions, using scRNA-seq dataset with annotated cell type information [8, 17]. These predefined proportions are then used as the ground truth fractions to assess the accuracy of deconvolution results. The pipeline incorporates the "heterogeneous" simulation strategy we proposed earlier [8], which is designed to reflect realistic biological variance among the simulated samples. Essential inputs for this process include raw scRNA-seq data (in non-log transformed scale and sourced from the same tissue as the reference) and cell-type/sample-ID labels of the single cells, which are necessary to guide the aggregation of cells and maintain appropriate biological variance within the simulated samples. The pipeline also supports other user-provided simulated data, provided that these samples are in non-log transformed scale and include ground truth cell type fractions. We recommend users to employ scRNA-seq datasets that are **different** from the one used to generate the reference, to

prevent information transfer from the reference to the bulk samples

2). Collection of real bulk samples with known proportions. While simulation serves as a potent tool for reference evaluation, incorporating real bulk samples is beneficial to fully account for the application on actual datasets. Our pipeline offers the option to integrate real bulk samples from TCGA cohort data for performance evaluations. Specifically, it applies the *build_tcga_obj()* function from the *deconvBenchmarking* [8] package, which downloads the expression data of a given tumor type from the xena browser [18] and automatically retrieved TCGA purity estimates from various methods [19] including ABSOLUTE [5], ESTIMATE [6], CPE [7] and LUMP [7]. The tumor purity estimates are used as proxies for malignant proportions, which will later be compared against the malignant estimations for performance evaluation.

3). Deconvolution & Performance evaluation Once bulk samples with known ground truth fractions are prepared, we can run deconvolution with user provided reference and directly visualize the results (Fig. S3).

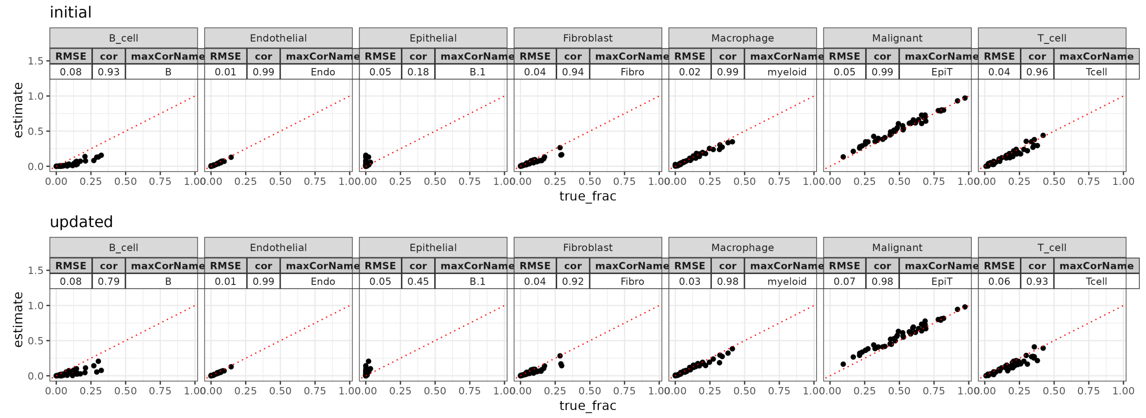

Figure S3: **An example of the performance visualization plot**. A representative scatter plot returned by the evolution pipeline showing the performance of the built-in CRC reference (CRC_refPhi) on simulated CRC samples. Two sets of plots are provided: “initial”, using scRNA-based reference, and “updated”, using the updated reference. For each cell type in the simulated bulk samples, the matched reference cell types are denoted by “maxCorName” and the performance metrics including Root Mean Square Error (RMSE) and Pearson correlation (cor) are provided.

The evaluation pipeline allows simultaneous assessment of multiple references and includes the option to include the “updated reference” (see Text S1 for details) as well. Considering the possible discrepancies between cell type names in the ground truth and the reference, the pipeline is designed to automatically identify pairs of matched truth-reference cell types based on the maximum Pearson correlation. The deconvolution performance, evaluated using Pearson correlation coefficients and RMSE (root mean square error) values between the ground-truth and estimated fractions, will then be documented and presented in a scatter plot (Fig. S3) for direct visualization.

The pipeline is now readily available at `https://github.com/humengying0907/InstaPrismSourceCode`, providing a convenient and user-friendly way for reference evaluation.

4. Recommendations on reference selection

After obtaining the performance data from the reference evaluation pipeline, we recommend the users to **1)** Choose a reference that contains a broad range of cell types. **2)** Select reference that excels in Pearson correlation and RMSE values for each cell type; and **3)** Select reference that consistently demonstrates good performance across the test datasets.

Again, we acknowledge that reference construction using scRNA-seq data remains an open challenge in the field of deconvolution, and there is yet no closed-form solution. Even for scRNA-seq datasets from the same tissue type, the resulting references can vary [20]. Various factors impact the consistency between references constructed from different datasets, including biological differences among samples (such as age, ethnicity and spatial heterogeneity), technical differences (such as labs, experimental protocols) and data processing differences (computational pipelines and cell-type annotation methods) [10]. When multiple references are available, one potential technique is to adopt a "consensus" deconvolution approach, which compares results from various references. Cell type fractions that show consistent results across references will be considered reliable, while those that vary significantly require extra attention.

To facilitate the reference construction process, we have provided a set of built-in references covering a broad range of cancer types (Table S1), following the guidance we provided above. The built-in reference is now readily available by the *InstaPrism_reference()* function, or can be downloaded directly from `https://github.com/humengying0907/InstaPrismSourceCode`.

Text S3: Impact of cell state specification on model performance

In the above section, we provided a general guideline for reference construction and introduced the evaluation pipeline to assess a reference's performance. However, in real practice, specifying cell states in the reference involves several methodological decisions, such as choosing a cell state clustering method, determining the granularity levels of cell states, and deciding which cell states to include in the reference. In this section, we will explore how different cell state specifications impact model performance and provide guidance on this hyperparameter setting.

1. Impact of duplicated reference

Consider the following example where we generated two identical columns for the Oligodendrocyte cell type in the reference matrix $A_{G\times S}$, using the built-in GBM reference (GBM_refPhi) from our package. Analyzing simulated GBM data (Table S1) with this reference, we observed that when duplicated cell states (Oligodendrocyte1 and Oligodendrocyte2) are present, their fraction estimates are identical. Each duplicated cell state accounts for a portion of the ground truth Oligodendrocyte fraction, and the sum of these fraction estimates matches the ground truth fraction levels (Fig. S4).

This indicates that in the presence of duplicated cell state information, the model adjusts the posterior estimates accordingly. As the model aggregates these estimates across cell states to produce the final cell type level posterior, the fraction estimates at cell type level stays robust. Experiments using other references also confirmed this observation (data not shown).

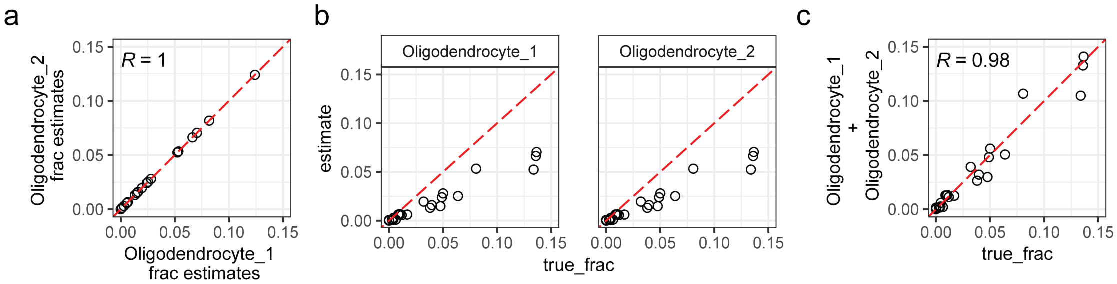

Figure S4: **Effect of duplicated reference. a** When duplicated cell states present in the reference (Oligodendrocyte1 and Oligodendrocyte2), fraction estimates for duplicated cell states are identical. **b** Duplicated cell states each explains part of the ground truth Oligodendrocyte fraction. **c** Fraction estimates from duplicate reference is additive, matching up with ground truth fraction levels.

This highlights the model's ability to handle redundancies in cell state specification. More importantly, this implies that even if very similar cell states are identified during the cell state clustering step, their fraction estimates are essentially **additive**. In fact, as long as the clustering captures the necessary heterogeneity (check next subsection for details), regardless of the specific clustering approach employed, the aggregated result remains reliable.

2. Impact of cell state granularity

Cell state granularity affects the resolution at which cellular differences are discerned and classified. The finer-grained the subclustering, the more cell states are identified and included in the reference. Theoretically, representing a cell type with different cell states captures diverse molecular characteristics present [21], which is especially valuable for heterogeneous groups like malignant cells. However, determining the optimal level of granularity is an unsupervised issue and does not have a closed-form solution.

With the increasing number of single cells analyzed in singe cell RNA sequencing, many scRNA-seq studies now provide not only cell type annotations but also finer-grained cell state annotations that reveal expression differences within cell types [22]. Utilizing cell state information from these original annotations provides a convenient way to specify the cell state granularity level.

Theoretically, other granularity levels exist as well. To explore how varying levels of granularity affects the performance of cell type deconvolution, we considered four levels of granularity:

- granularity_v0: all cell types are represented as a single profile without further subclustering
- granularity_v1: only the malignant populations are subjected to subclustering
- granularity_v2: granularity level provided by the scRNA studies, with major cell types being subclustered and labeled according to their transcriptional profiles (the same level as in InstaPrism built-in reference)
- granularity_v3: cell types are being reclustered using the get_subcluster() function from InstaPrism package, with a finer-grained granularity level compared to granularity_v2

Building reference with various granularity levels for cell states and applying it to simulated data (Table S1), we observed that **i)** specifying no subclustering information (granularity_v0) or including

subclustering information (granularity_v1) in malignant cells only generally results in poorer performance; **ii)** adding finer-grained information (granularity_v3) on top of the granularity level provided by the scRNA studies does not yield additional benefits for deconvolution performance, and **iii)** the subclustering method is not the dominant factor in performance, as cell states specified with different methods (granularity_v2 and granularity_v3) exhibit similar performance.

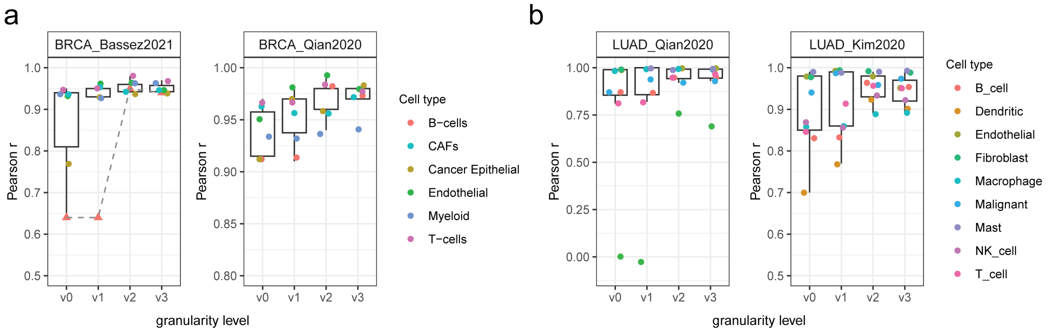

Figure S5: **Impact of cell state granularity.** Boxplot showing the performance of the reference with different granularity levels in four simulated datasets, evaluated by the Pearson correlation between estimated and ground truth fractions for each reference cell type. In **a**, references are constructed using the same scRNA data used for BRCA_refPhi construction. From granularity level v0 to v3, the number of cell states increases: v0 (n_cs = 8), v1 (n_cs = 39), v2 (n_cs = 76), and v3 (n_cs = 90). In **b**, references are constructed using the same scRNA data used for LUAD_refPhi construction, with v0 (n_cs = 13), v1 (n_cs = 36), v2 (n_cs = 77), and v3 (n_cs = 92). Specifically, reference with granularity_v2 is the InstaPrism built-in reference.

This indicates with subclustering information specified in both malignant and non-malignant cells (as done in granularity_v2), transcriptional difference within cell types are properly maintained, which is beneficial for accurate cell type fraction prediction. The results also align with our previous findings that duplicated cell state has a completely additive effect, as adding more cell states (granularity_v3) does not notably alter the deconvolution results.

We note that here we do not explicitly recommend the subclustering methods or the exact number of cell states to be used. The general principle is to use any subclustering method, as long as it can discern cellular differences across both malignant and non-malignant cells. For tumor types with known molecular subtypes, such as breast cancer, which includes HER2-enriched, Luminal A, Luminal B, and Basal-like subtypes [23], the malignant cell states should encompass these major subtypes. For non-malignant cell types such as lymphoid and myeloid cells, subclustering is highly recommended to capture potential molecular differences and enhance deconvolution performance. When a comparison of granularity levels is needed, one can refer to our evaluation pipeline for direct assessment.

3. Impact of missing cell types

Another challenge in reference construction is the “missing cell type” problem, where certain cell types present in the bulk samples are absent from the reference. This issue typically occurs with rare or low-frequency cell types that are not adequately recognized and documented in the scRNA-seq datasets used for reference construction [24]. The direct consequence to this is

obvious: there will be no fraction estimation for the missing cell types. Moving forward, to explore the impact on the prediction of the remaining cell types, we will consider the following examples:

Using the InstaPrism built-in LUAD reference and a simulated lung cancer dataset (Table S1), we gradually remove one cell type at a time from the reference, starting with the least abundant cell types in the simulated bulk. The process continued until the reference contained only four major cell types: malignant cells, macrophages, T cells and B cells. Note that during this process, the cell types present in the reference retained the same cell state information. In Fig. S6, we observed that despite this reduction, the fraction prediction for the remaining cell types remains robust. Even with the last reference, the Pearson correlation for the cell types was still around 0.92. Experiments using other references also confirmed this observation (data not shown).

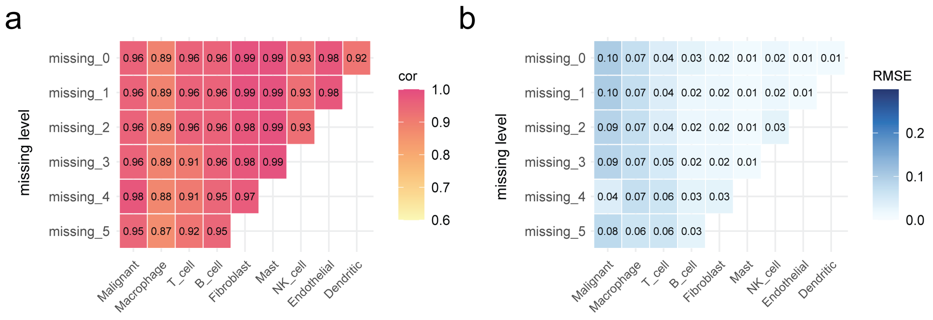

Figure S6: **Effect of missing cell types in the reference**. Heatmap showing the deconvolution performance using reference with varying levels of missing cell types on simulated LUAD samples. The performance is evaluated by **a** Pearson correlation and **b** RMSE values between the estimated and ground truth fractions of the remaining cell types. Each row corresponds to a different reference configuration, ranging from no missing cell types to five missing cell types.

We note that in the above testing we focus exclusively on using the InstaPrism built-in reference, which primarily targets the tumor microenvironment. In addition, when removing cell types from the reference, the remaining cell types retain the same cell state information. However, in a separate testing environment when the above conditions are not met, different results may be observed. For example, in Ivich [24]' s study, where they tested the effect of missing cell types in simulated PBMC (peripheral blood mononuclear cells) samples using a reference constructed from the PBMC3k [25] dataset, worse deconvolution results were observed for the remaining cell types. This could be because the reference they used was not properly optimized and did not include sufficient cell state information, or because PBMC cells from health donors are intrinsically less diverse than those in the tumor microenvironment [26], making them more vulnerable to the missing cell-type problem.

Overall, our empirical tests indicate that missing cell types do not significantly hinder predictions for the remaining cell types. However, we still recommend using scRNA-seq data that encompasses a broad range of cell types for reference construction to achieve a more accurate representation of the tissue microenvironment.

4. Selection of cell types in the reference

The advancement of single-cell technology has helped uncover the complex tissue microenvironment, including rare cell types [27] and distinct cell subsets [22]. Utilizing these finely annotated scRNA datasets for reference construction now presents a new challenge: whether to include all identified cell types. Typically, deconvolution performance for rare cell types is difficult to assessed experimentally using simulated data, as scRNA data used for bulk simulations may not include these new cell types or might fail to identify them. For this issue, we consider both including and excluding rare cell types as feasible options. The rationale for inclusion is that it could enhance the representation of tissue microenvironment, whereas the rationale for exclusion is that removing these cell types does not impact the prediction for other cell types, as we have demonstrated in the previous subsection, and rare cell types typically exhibit a low frequency (less than 0.1%) in deconvolution results (Fig. S7), suggesting that they may not present meaningful compositional differences.

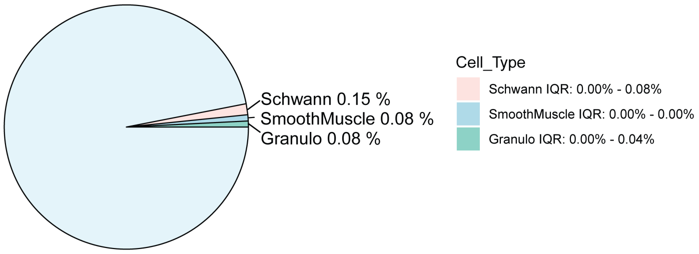

Figure S7: **An example of rare cell type deconvolution results.** A pie plot showing the average fraction estimates of three rare cell types from 689 TCGA-CRC samples, using the built-in CRC reference (CRC_refPhi). For visibility, the labeled segmentations in the pie plot are scaled up 10 times and are not proportional to the actual percentages. The interquartile range (IQR), indicating the 25-75% range of these fraction estimates, is labeled in the legend.

In InstaPrism built-in reference, we choose to include all cell types annotated from the scRNA data to ensure a comprehensive representation of the tumor microenvironment (Table S2). Note that with the flexibility of reference construction in InstaPrism, users can customize these references by removing or modifying different cell types as needed. For example, a rare cell type that consistently exhibits low frequency (less than 0.1%) in the provided bulk data and external bulk datasets like TCGA can be excluded from the reference. Additionally, if one is interested in estimating the overall fraction of myeloid populations, they can merge cell states associated with all myeloid cells, such as macrophages, monocytes, and dendritic cells into a new cell type in the reference to obtain the overall fraction estimates. Alternatively, if one is interested in a specific population of cell type, such as CD4 T cell fractions, they can isolate cell states associated with CD4 T cells and categorize them as a separate cell type within the reference to obtain associated fraction estimates. Together, this flexibility enhances the applicability of InstaPrism built-in reference and can be further generalized to other user-constructed references.

Text S4: Model convergence

Similar to BayesPrism, InstaPrism essentially optimizes the log likelihood of the underlying topic model. As the InstaPrism algorithm removed the Dirichlet prior α in the modeling, the formulation of the likelihood has been simplified from the original likelihood derived in BayesPrism. Specifically, consider the following notations:

- Prior: $A_{G\times S}$: a gene by cell-state reference matrix, with $\sum_g A_{g,s} = 1$ for all cell state $s \in [1, S]$
- Prior: $X_{G\times N}$: input bulk samples for deconvolution analysis, with genes in rows and samples in columns
- Posterior: $\theta_{N\times S}$: cell state fraction estimates, with $\sum_s \theta_{,s} = 1$ for each sample $n \in [1, N]$
- Posterior: $Z_{G\times N\times S}$: cell state-specific gene expression estimates, with $\sum_{s=1}^{S} Z_{gns} = X_{gn}$ for each gene $g \in [1, G]$

The likelihood of the model can now be expressed as:

$$p(\theta, Z|A, X) \propto \prod_{n=1}^{N}\{\prod_{g=1}^{G}\prod_{s=1}^{S}(A_{gs} \circ \theta_{ns})^{Z_{gns}}\}$$

We note that the log likelihood function is directly influenced by the model's complexity, including factors such as number of bulk samples, number of genes and number of cell states in the reference. Over iterations, the model will optimize the posterior estimates until the log likelihood no longer improves. This is an intrinsic feature of topic modeling and does not depend on hyperparameter choices in the model.

To illustrate this, we plotted the log likelihood changes over iterations using a series of eight models on a simulated breast cancer dataset (Fig. S8). Model 1 utilizes the complete built-in BRCA_refPhi, while each subsequent model (Models 2 to 8) omits one cell type from the BRCA_refPhi at a time. Omitting a cell type means excluding all the associated cell states of that type. This process results in progressively reduced model complexity for Models 2 through 8. Although these models exhibit an increasing "missing cell type" problem, all models eventually converge, with simpler models tending to converge earlier. This suggests that the log likelihood is suitable only for monitoring convergence within the same model and cannot be used for direct comparisons between different models, as it is mainly impacted by the model's complexity.

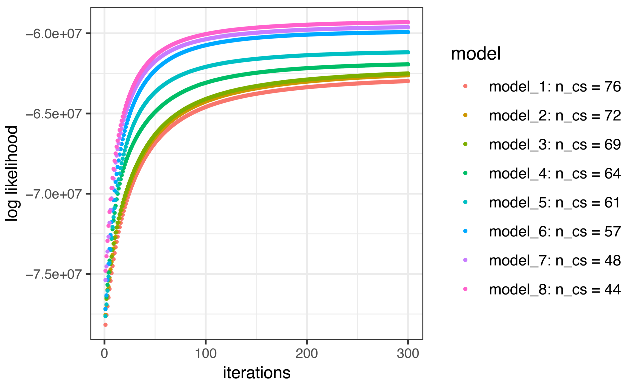

Figure S8: **Convergence of log likelihood over iterations.** Each curve represents a deconvolution process with different number of cell types included in the reference, when running deconvolution on a simulated breast cancer dataset.

We note that calculating log likelihood over iterations is computational intensive, therefore we have included this only as a supplementary feature in our source code for interested users [28]. In the main deconvolution function *InstaPrism()*, We implemented another simpler approach for convergence control. This involves calculating the absolute difference ("abs-difference") in fraction estimates between the penultimate and final iterations as an indicator of convergence. A high "abs-difference" value suggests that the model is still updating the fraction estimates and the log likelihood has not yet converged, whereas a small "abs-difference" value indicates that the model has likely reached convergence. Specifically, if the abs-difference for any cell type in any sample exceeds 0.01, the function will issue a warning and promote selecting a higher number of iterations. We have also incorporated an "abs-difference" value visualization feature (enabled by setting *convergence.plot = TRUE*) that aids in the monitoring of the model's convergence status.

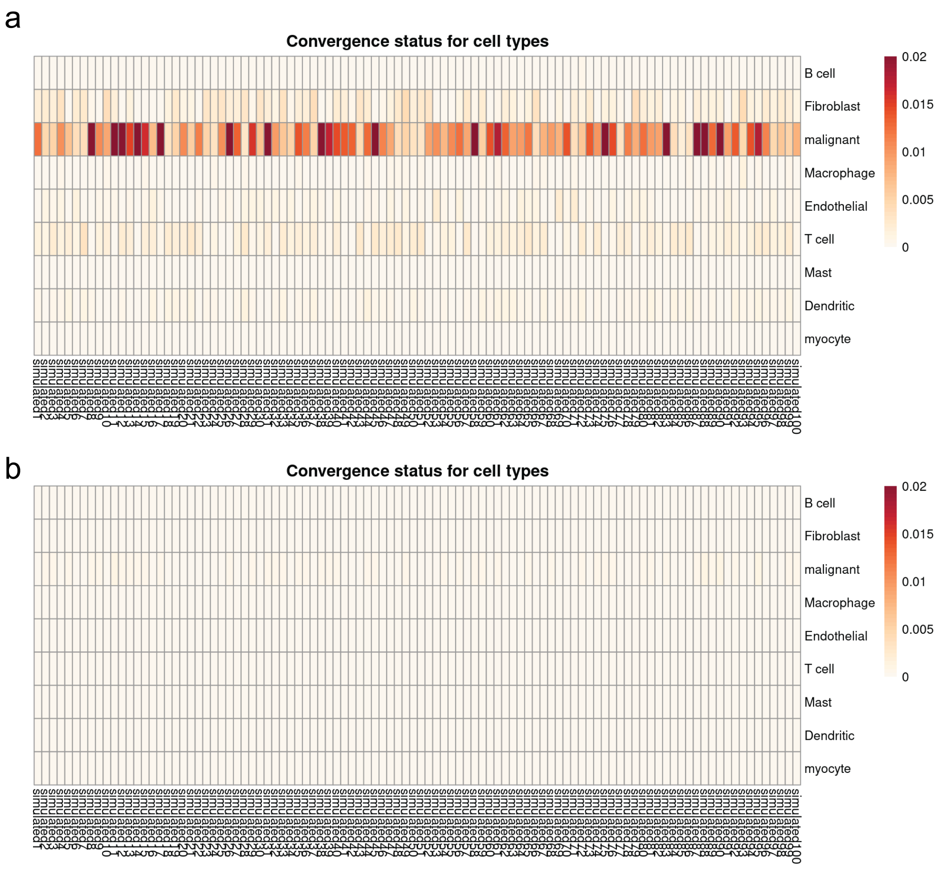

Figure S9: **An example of convergence plot from the *InstaPrism()* function.** Heatmap comparing the absolute difference in fraction estimates between the penultimate and final iterations for all input samples, as an indicator of model convergence. Scenario **a** displays a significant difference in fraction estimates due to insufficient iterations (*n.iter = 20*), indicating a lack of model convergence, whereas scenario **b** presents negligible differences in fraction estimates across samples with sufficient iterations (*n.iter = 100*), suggesting that the model no longer updates fraction estimates and has thus achieved convergence.

Taking an example from the InstaPrism tutorial (`https://humengying0907.github.io/InstaPrism_tutorial.html`): when the number of iterations is insufficient (as shown in Fig.

S9a), the convergence checking heatmap will display high values of “abs-difference,” prompting a warning to consider increasing the number of iterations. Conversely, with a higher number of iterations (as in Fig. S9b), the “abs-difference” becomes negligible across samples, indicating that the model no longer updates the fraction estimates and has thus achieved convergence.

Text S5: Construction & Validation of InstaPrism Built-in reference

To streamline the deconvolution process, we have provided a set of built-in references covering a broad range of cancer types, adhering to the guidelines for reference construction we proposed (see Text S2 for details). Specifically, we selected seven recent and large scale scRNA studies that provides extensive annotations at both the cell type and cell state levels (Table S1). The original cell type and cell state annotations are used to guide the cell state specification step during reference construction. For malignant cell types lacking cell state annotations, we either reclustered the cell types using the *quick_cluster()* function or relied on their source of origin (patient identifiers) to specify the cell states. Ribosomal and mitochondrial genes are removed from the reference following guidance from BayesPrism. All the reference are generated using the *refPrepare()* function in InstaPrism. The details of the cell type and cell state information in each built-in reference are listed in Table S2.

1. Validation using simulated data

To validate the performance of these built-in references, we applied the evaluation pipeline we proposed earlier (see Text S2.3 for details). Specifically, we simulated bulk samples using scRNA-seq datasets that are **different** from the one used to generate the scRNA-based references. This approach ensures robust reference validation, as no information from the reference is used to generate the simulated bulk samples, mirroring real-world deconvolution scenarios when there are inevitable technical differences between the reference data and the bulk samples being analyzed [10]. Details of the validation scRNA datasets used for bulk simulation can be found in Table S1.

Each simulated bulk dataset contains a total of 50 simulated samples, which comprise two parts: 1) pseudo-bulk samples created by aggregating cells from the same biological sample, and 2) heterogeneously simulated bulk samples, created using the heterogeneous bulk simulation strategy we proposed recently [8], a simulation method specifically designed to preserve appropriate sample-level heterogeneity in the simulated samples. In the pseudo-bulk samples, cell type fractions reflect the original distributions within the biological samples, whereas in the heterogeneously-simulated samples, fractions are drawn from an asymmetric Dirichlet distribution, with the α parameter determined by cell type frequencies from the source scRNA-seq dataset, and the shape parameter adjusted accordingly to ensure a proper fraction range.

Applying the InstaPrism built-in reference to the validation datasets, we first got a set of estimated fraction matrices, denoted as $\theta_{50\times k}$, where each column corresponds to a cell type present in the reference. Given that the simulated bulk samples are generated from a different scRNA-seq dataset than the one used for reference construction, the ground truth fraction matrices, $\theta_{50\times k'}$, do not necessarily have the same column names or the same number of columns as the estimated fraction matrices. This discrepancy is important for testing the generalizability of the reference. The matching process between reference and ground truth cell types is facilitated by finding the maximum Pearson correlation between the two sets of fraction matrices and manually adjusted to include only cell types present in both sets.

The reference performance was then assessed using Pearson correlation between the ground truth and estimated fractions for each matched cell type. In Fig. S10, we summarized the Pearson correlation and plotted it against the average fraction of each cell type in the simulated samples. As previously explained, the fractions in these samples are designed to mirror the actual abundance distributions of cell types in the scRNA data used for the bulk simulation. Therefore, the average cell type fraction (x axis) in this context serves as a proxy for the typical cell type abundance in the tumor tissue. A detailed scatter plot comparing the estimated and ground truth cell type fractions in the validation dataset can be found at Fig. S12.

Note that for this evaluation we focus exclusively on the results from the built-in reference-based deconvolution, without the “reference update” step (Text S1). Across the validation datasets, the estimated fractions closely align with the ground truth fractions. with an average Pearson correlation of 0.92 per cell type. Again, we highlight that this performance is observed on bulk samples that are simulated without borrowing any known cellular information from the built-in reference and are designed to exhibit not merely proportional differences, but also appropriate sample-level heterogeneity [8]. Although these features represent typical challenges in real-world deconvolution tasks [8], the InstaPrism algorithm, combined with the built-in reference, achieves high performance, highlighting the robustness of the algorithm and the generalizability of the built-in references.

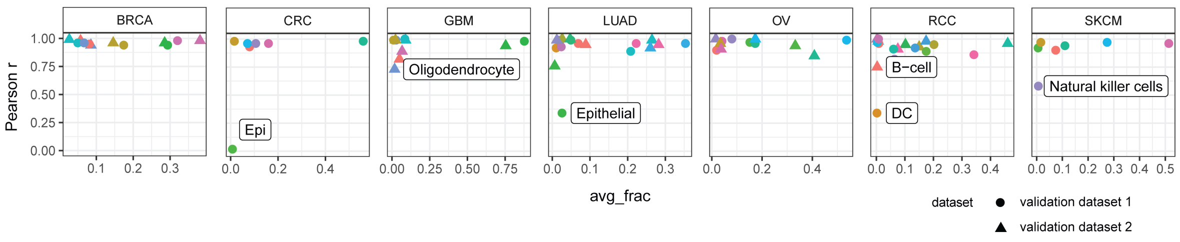

Figure S10: **Performance of InstaPrism built-in reference on simulated data.** Scatter plot showing the performance of each cell type reference against the relative abundance of the cell type in simulated datasets. The x-axis represents the average fraction of each cell type, and the y-axis corresponds to the Pearson correlation coefficient between the estimated and ground truth cell type proportions. Different colors correspond to different cell types from the reference, while different shapes denote different validation datasets used under the same reference. Cell types with lower performance (correlation < 0.75) are indicated with text labels.

We observed three instances of notably lower performance (correlation < 0.5) in our validation datasets: the CRC-epi, LUAD-epithelial, and RCC-dc signatures. Two of these instances involve normal epithelial cells and one involves dendritic cells. Both cell types are typically present in low frequencies in bulk samples and are usually identifiable only at single-cell resolution. We note that it is possible the scRNA-seq datasets used for reference construction and bulk simulation may have applied different criteria for labeling these cell types. Therefore, the predicted signatures are not align with the established ground truths. Indeed, normal epithelial cells often have overlapping signatures with cancerous epithelial cells, and dendritic cells are frequently found to be mixed the myeloid cells in low-dimensional representations such as UMAP [22, 29]. We suggest that these instances of low performance do not necessarily indicate incorrect signatures in the built-in reference, but rather highlight the challenges in accurately representing these cell types in general. Given that these cell types are typically infrequent, exhibit minimal compositional differences, and

do not affect the deconvolution results of other cell types, it may be advisable to exclude them from the reference in real practice.

Additionally, two other signatures—GBM-oligodendrocyte and RCC-Bcell—show moderate performance (correlation < 0.75). Note that this occurs in one validation dataset but not in others, which may again be due to inconsistent labeling in the lower-performing validation dataset. For these signatures, we recommend retaining them in the reference as they still serve as useful guides for understanding the compositional information of the bulk samples.

2. Validation using TCGA data

We also incorporated real bulk samples from the TCGA cohort data (Table S3) to further assess the performance of the built-in reference. Following our proposed evaluation pipeline, we downloaded TCGA gene expression data along with associated tumor purity estimates [5, 6, 7]. These tumor purity estimation methods, originally developed to measure the proportion of tumor cells from the samples [30], were designed to provide insight into the tumor microenvironment and have been shown to be important in genomic analyses, such as tumor mutation burden [31] and intratumor heterogeneity estimation [32]. In our validation, we used these estimates as proxies to ground truth malignant proportions.

In Fig. S11a, we summarized the performance of each built-in reference by calculating the correlation between the malignant estimates and various TCGA tumor purity estimates. As an extension, we also included other deconvolution methods including CIBERSORTx [33], EPIC [34], DeconRNASeq [35], and DeMiXT [36] in the comparison. The malignant fraction estimates from these methods were obtained from Revkov et al. [30]; in their work, they applied these deconvolution methods to TCGA samples and reported the malignant fraction estimates specifically.

Across various measures of TCGA tumor purity, the malignant fraction estimates from InstaPrism using the built-in reference, generally reflect tumor purity estimates and consistently rank at the top compared to other methods. We note that since different purity estimation methods utilize different modalities [7] of data and follow distinct underlying estimation criteria, the resulting purity estimates should only be considered as approximations, not the definitive ground truth measurements.

For transcription-based purity methods ESTIMATE [6], which use the same modality data—gene expression data—as that used in deconvolution analyses, all methods demonstrate better correlation compared to other purity estimation methods, with InstaPrism achieving an average Pearson correlation of 0.79 across different tumor types. For the somatic copy-number alteration-based method ABSOLUTE [5], InstaPrism exhibits an average Pearson correlation of 0.6, surpassing other advanced methods. For other TCGA purity estimation methods such as CPE [7] and LUMP [7], InstaPrism with the built-in reference generally achieves moderate correlation. Less association was observed for IHC, an image-based purity estimation method, across all deconvolution methods; this is consistent with previous findings that gene expression-based estimates of malignancy typically correlate poorly with IHC-based purity measures [1].

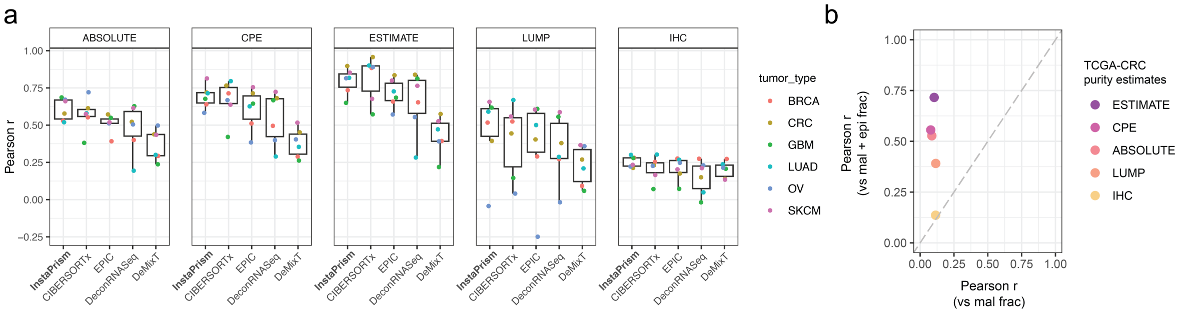

Figure S11: **Performance of InstaPrism built-in reference on TCGA purity estimation. a** Boxplot comparing the performance of different deconvolution methods in estimating TCGA tumor purity, measured by Pearson correlation between the estimated malignant proportion and the known TCGA tumor purity quantities. Each dot represents a tumor type and each panel of the figure corresponds to a specific TCGA tumor purity estimation method. **b** Scatter plot comparing the correlation values when comparing tumor purity estimates with malignant proportion estimates versus malignant plus normal epithelial proportions estimates in TCGA RCC samples, using the built-in RCC reference (RCC_refPhi), with each dot corresponding to a different TCGA purity estimation method.

Overall, the malignant fraction estimates using the built-in reference properly reflect TCGA tumor purity estimates, suggesting its suitability for tumor purity estimation purposes in facilitating genomic analyses such as tumor mutation burden [31] and intratumor heterogeneity estimation [32].

We note that the TCGA-RCC dataset is a significant outlier, with the estimated malignant signature poorly associated with any TCGA purity estimates (cor < 0.2, Fig. S11b). We hypothesized that the purity estimates being compared do not accurately reflect the cancerous epithelial cells but also include normal epithelial cells misclassified as malignant. Indeed, when we compared the combined fractions estimates for both malignant and normal epithelial cells, the correlation showed a significant improvement. Therefore, we recommend exercising caution when using purity estimates from the TCGA-RCC dataset in future analyses.

Together, we have demonstrated good performance and generalizability of the InstaPrism built-in reference by validating it using both the simulated and real bulk samples. The reference captures comprehensive insights into the cellular components of the underlying tumor microenvironment, offering a ready-to-use option for the bulk deconvolution task. To reproduce these results or to test with your own data, please refer to our source code at `https://github.com/humengying0907/InstaPrismSourceCode`.

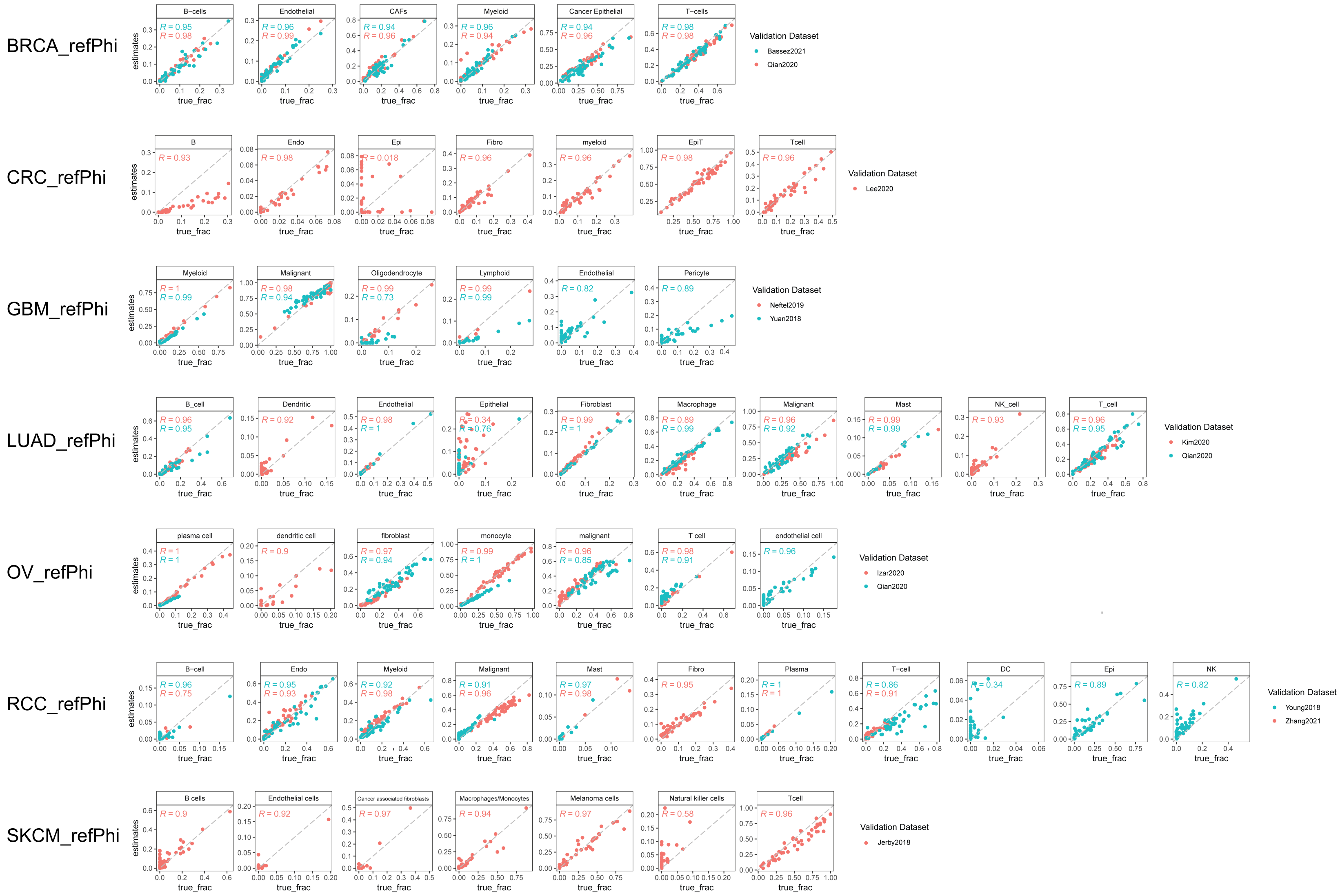

Figure S12: **Detailed deconvolution results from validation datasets.** Scatter plot comparing ground truth fraction (x axis) and fraction estimates (y axis) for matched cell-types in each validation dataset, using InstaPrism built-in reference.

Supplementary Tables

Table S1: scRNA data used for reference construction and bulk simulation

Table S1: scRNA data used for reference construction and bulk simulation

reference	**source scRNA**	#cells	source platform	**validation dataset**[1]	#cells	validation platform	related content [2]
BRCA_refPhi	Wu et al. 2021 [37]	100,064	10X v2, 3' & 5'	Bassez et al. 2021 [38]	48.568	10X 5' kit	Text S1, S3.2, S5
				Qian et al. 2020 [39]	16, 537	10X v2, 5' kit	Text S1, S3.2, S4, S5
CRC_refPhi	Pelka et al. 2021 [22]	371,223	10X 3' kit	Lee et al. 2020 [40]	21,657	10X v2, 3' kit	Text S1, S2.3, S5
GBM_refPhi	Ruiz et al. 2022 [27]	338,564	mixted platforms[3]	Yuan et al. 2018 [41]	23,793	Microwell array-based platform	Text S1, S5
				Neftel et al. 2019 [42]	24, 131	SmartSeq2	Text S1, S3.1, S5
LUAD_refPhi	Xing et al. 2021 [43]	118,293	10X v2, 3' kit	Qian et al. 2020 [39]	27,262	10X v1/v2, 3' kit	Text S1, S3.2, S5
				Kim et al. 2020 [44]	32,493	10X v2, 3' kit	Text S1, S3.2, S3.3, S5
OV_refPhi	Vazquez et al. 2022 [45]	929,690	10X v3, 3' kit	Qian et al. 2020 [39]	16,951	10X v2, 3' kit	Text S1, S5
				Izar et al. 2020 [46]	10,788	10X v2, 3' kit	Text S1, S5
RCC_refPhi	Li et al. 2022 [29]	270,855	10X 5' kit	Zhang et al. 2021 [47]	29, 474	10X v2, 3' kit	Text S1, S5
				Young et al. 2018 [48]	125,139	10X	Text S1, S5
SKCM_refPhi	Tirosh et al. 2016 [49]	4,645	SmartSeq2	Jerby et al. 2018 [50]	7,186	SmartSeq2	Text S1, S5

1: validation scRNA-seq datasets from the same cancer type are used to generate simulated bulk samples, each containing 50 simulated bulk samples
2: bulk samples simulated from the validation dataset are associated with these sections
3: GBM_refPhi is constructed from a harmonized single-cell GB core reference [27], comprising 16 scRNA-seq studies across multiple platforms

Table S2: Overview of cell type and cell state information in built-in reference

BRCA_refPhi

Cell Types	Cell States
Endothelial	Endothelial ACKR1
	Endothelial RGS5
	Endothelial CXCL12
	Endothelial Lymphatic LYVE1
CAFs	CAFs MSC iCAF-like s1
	CAFs MSC iCAF-like s2
	CAFs Transitioning s3
	CAFs myCAF like s4
	CAFs myCAF like s5
PVL	PVL Differentiated s3
	PVL_Immature s2
	PVL Immature s1
	Cycling PVL
B-cells	B cells Memory
	B cells Naive
	Plasmablasts
T-cells	T_cells_c4_CD8+_ZFP36
	T_cells_c6_IFIT1
	T_cells_c5_CD8+_GZMK
	T_cells_c7_CD8+_IFNG
	T_cells_c8_CD8+_LAG3
	T_cells_c0_CD4+_CCR7
	T_cells_c1_CD4+_IL7R
	T_cells_c2_CD4+_T-regs_FOXP3
	T_cells_c3_CD4+_Tfh_CXCL13
	T_cells_c9_NK_cells_AREG
	T_cells_c11_MKI67
	T_cells_c10_NKT_cells_FCGR3A
Myeloid	Myeloid_c10_Macrophage_1_EGR1
	Myeloid_c12_Monocyte_1_IL1B
	Myeloid_c2_LAM2_APOE
	Myeloid_c1_LAM1_FABP5
	Myeloid_c8_Monocyte_2_S100A9
	Myeloid_c7_Monocyte_3_FCGR3A
	Myeloid_c9_Macrophage_2_CXCL10
	Cycling_Myeloid
	Myeloid_c5_Macrophage_3_SIGLEC1

BRCA_refPhi (continued)

Cell Types	Cell States
	Myeloid_c11_cDC2_CD1C
	Myeloid_c4_DCs_pDC_IRF7
	Myeloid_c3_cDC1_CLEC9A
	Myeloid_c0_DC_LAMP3
Normal Epithelial	Myoepithelial
	Luminal Progenitors
	Mature Luminal
Cancer Epithelial	Cancer Cycling_1
	Cancer Her2 SC_5
	Cancer Her2 SC_1
	Cancer LumB SC_2
	Cancer Cycling_8
	Cancer Her2 SC_2
	Cancer Her2 SC_3
	Cancer LumB SC_3
	Cancer LumB SC_4
	Cancer Cycling_4
	Cancer Basal SC_8
	Cancer LumA SC_2
	Cancer LumA SC_4
	Cancer Her2 SC_4
	Cancer Basal SC_4
	Cancer Cycling_3
	Cancer Basal SC_2
	Cancer Basal SC_3
	Cancer LumB SC_1
	Cancer Cycling_6
	Cancer Basal SC_1
	Cancer Her2 SC_6
	Cancer Cycling_2
	Cancer Basal SC_7
	Cancer Cycling_7
	Cancer Cycling_5
	Cancer Basal SC_5
	Cancer Basal SC_6
	Cancer LumA SC_1
	Cancer LumA SC_5

BRCA_refPhi (continued)

Cell Types	Cell States
	Cancer LumB SC_5
	Cancer LumA SC_3

CRC_refPhi

Cell Types	Cell States
EpiT	Tumor cE01 (Stem/TA-like)
	Tumor cE02 (Stem/TA-like/Immature Goblet)
	Tumor cE03 (Stem/TA-like prolif)
	Tumor cE04 (Enterocyte 1)
	Tumor cE05 (Enterocyte 2)
	Tumor cE06 (Immature Goblet)
	Tumor cE07 (Goblet/Enterocyte)
	Tumor cE08 (Goblet)
	Tumor cE09 (Best4)
	Tumor cE10 (Tuft)
	Tumor cE11 (Enteroendocrine)
Peri	cS17 (Pericyte)
	cS15 (Pericyte)
	cS18 (Pericyte)
	cS19 (Pericyte)
	cS20 (Pericyte prolif)
	cS16 (Pericyte)
DC	cM09 (mregDC)
	cM04 (DC2)
	cM05 (DC2 C1Q+)
	cM06 (DC IL22RA2)
	cM03 (DC1)
	cM07 (pDC)
	cM08 (AS-DC)
NK	cTNI22 (cTNI22)
	cTNI24 (NK GZMK+)
	cTNI25 (NK XCL1+)
	cTNI23 (NK CD16A+)
Endo	cS13 (Endo venous-like)
	cS08 (Endo arterial-like)
	cS02 (Endo capillary)
	cS12 (Endo)
	cS04 (Endo)
	cS11 (Endo proif)
	cS09 (Endo)
	cS10 (Endo tip cells)
	cS01 (Endo arterial)

CRC_refPhi (continued)

Cell Types	Cell States
	cS14 (Endo)
	cS05 (Endo venous)
	cS06 (Endo lymphatic)
	cS03 (Endo capillary)
	cS07 (Endo capillary-like)
Fibro	cS27 (CXCL14+ CAF)
	cS29 (MMP3+ CAF)
	cS25 (Fibro CCL8+)
	cS28 (GREM1+ CAF)
	cS24 (Fibro BMP-producing)
	cS30 (CAF CCL8 Fibro-like)
	cS22 (Fibro stem cell niche)
	cS21 (Fibro stem cell niche)
	cS23 (Fibro BMP-producing)
	cS26 (Myofibro)
	cS31 (CAF stem niche Fibro-like)
Epi	cE01 (Stem/TA-like)
	cE03 (Stem/TA-like prolif)
	cE02 (Stem/TA-like/Immature Goblet)
	cE08 (Goblet)
	cE11 (Enteroendocrine)
	cE04 (Enterocyte 1)
	cE10 (Tuft)
	cE05 (Enterocyte 2)
	cE06 (Immature Goblet)
	cE09 (Best4)
	cE07 (Goblet/Enterocyte)
B	cB1 (B IGD+IgM+)
	cB2 (B GC-like)
	cB3 (B CD40+ GC-like)
	cP2 (Plasma IgG)
	cP1 (Plasma IgA)
	cP3 (Plasma IgG prolif)
Mast	cMA01 (Mast)
Granulo	cM10 (Granulocyte)
ILC	cTNI26 (ILC3)
SmoothMuscle	cS32 (Smooth Muscle)
Schwann	cS33 (Schwann)

CRC_refPhi (continued)

Cell Types	Cell States
myeloid	cM02 (Macrophage-like)_1
	cM02 (Macrophage-like)
	cM01 (Monocyte)
Tcell	cTNI05 (CD4+ IL17+)
	cTNI04 (CD4+ IL7R+CCL5+)
	cTNI09 (CD4+ Treg prolif)
	cTNI08 (CD4+ Treg)
	cTNI02 (CD4+ IL7R+SELL+)
	cTNI03 (CD4+ IL7R+HSP+)
	cTNI06 (CD4+ TFH)
	cTNI01 (CD4+ IL7R+)
	cTNI07 (CD4+ CXCL13+)
	cTNI11 (CD8+GZMK+)
	cTNI10 (CD8+ IL7R+)
	cTNI13 (CD8+ T IL17+)
	cTNI16 (CD8+ CXCL13+ prolif)
	cTNI14 (CD8+ CXCL13+)
	cTNI12 (CD8+ IL7R+)
	cTNI15 (CD8+ CXCL13+ HSP+)
	cTNI20 (PLZF+ T)
	cTNI21 (PLZF+ T prolif)
	cTNI18 (gd-like T PDCD1+)
	cTNI17 (gd-like T)
	cTNI19 (gd-like T prolif)

GBM_refPhi

Cell Types	Cell States
Oligodendrocyte	Oligodendrocyte
Pericyte	Pericyte
Neuron	Neuron
Astrocyte	Astrocyte
Endothelial	Endo arterial
	Tip-like
	Endo capilar
Myeloid	TAM-BDM hypoxia/MES
	cDC2
	TAM-MG aging sig
	TAM-BDM anti-infl
	Mono anti-infl
	Mono hypoxia
	Mono naive
	TAM-MG pro-infl I
	TAM-BDM MHC
	TAM-MG pro-infl II
	TAM-MG prolif
	TAM-BDM INF
	Mast
	DC3
	DC1
	DC2
	cDC1
	pDC
Lymphoid	Stress sig
	Prolif T
	CD8 EM
	CD8 NK sig
	Reg T
	CD8 cytotoxic
	B cell
	CD4 rest
	CD4 INF
	Plasma B
	NK
Malignant	MES-like hypoxia independent_1
	OPC-like_1
	AC-like_5
	AC-like_2

GBM_refPhi (continued)

Cell Types	Cell States
	MES-like hypoxia/MHC_2
	NPC-like neural_1
	OPC-like Prolif_1
	AC-like Prolif_1
	MES-like hypoxia/MHC_3
	NPC-like OPC_1
	NPC-like Prolif_1
	NPC-like OPC_2
	AC-like_6
	OPC-like_2
	MES-like hypoxia/MHC_1
	AC-like_4
	AC-like_3
	AC-like_1
	OPC-like_3
OPC	oligodendrocyte precursor cell
RG	radial glial cell

LUAD_refPhi

Cell Types	Cell States
T_cell	CD4-C1
	CD4-C2
	CD8-C1
	CD8-C2
	CD8-C4
	CD4-C3
	CD4-C4
	CD8-C3
	CD8-C5
	CD4-C5
NK_cell	NK_cell_Normal_Lung
	NK_cell_Subsolid_Nodule
	NK_cell_Primary_LUAD_with_Met
Macrophage	Macrophage_Normal_Lung_4
	Macrophage_Normal_Lung_3
	Macrophage_Normal_Lung_2
	Macrophage_Normal_Lung_1
	Macrophage_Subsolid_Nodule_3
	Macrophage_Subsolid_Nodule_1
	Macrophage_Subsolid_Nodule_2
	Macrophage_Primary_LUAD_with_Met_1
Monocyte	Monocyte_Normal_Lung
	Monocyte_Subsolid_Nodule
	Monocyte_Primary_LUAD_with_Met
Dendritic	DCs-C2
	pDCs
	DCs-C3
	DCs-C1
Epithelial	AT1
	AT2
	Basal
	EPCAM+ cells
	Club
	Ciliated
Malignant	Malignant_SSN05
	Malignant_SSN09
	Malignant_SSN10
	Malignant_SSN11
	Malignant_SSN12
	Malignant_SSN19

LUAD_refPhi (continued)

Cell Types	Cell States
	Malignant_SSN21
	Malignant_SSN22
	Malignant_SSN24
	Malignant_SSN25
	Malignant_SSN27
	Malignant_SSN28
	Malignant_SSN29
	Malignant_SSN30
	Malignant_SSN31
	Malignant_SSN33
	Malignant_NM3C
	Malignant_NM3M
	Malignant_NM3E
	Malignant_NM4C
	Malignant_NM6C
	Malignant_NM6E
	Malignant_NM4M
	Malignant_NM4E
Granulocytes	Granulocytes
B_cell	B_cell_Normal_Lung
	B_cell_Subsolid_Nodule
	B_cell_Primary_LUAD_with_Met
	B_cell_0
	B_cell_1
	B_cell_2
	B_cell_3
Endothelial	Endothelial_Normal_Lung
	Endothelial_Subsolid_Nodule
	Endothelial_Primary_LUAD_with_Met
Mast	Mast_Normal_Lung
	Mast_Subsolid_Nodule
	Mast_Primary_LUAD_with_Met
Fibroblast	Fibroblast_Normal_Lung
	Fibroblast_Subsolid_Nodule
	Fibroblast_Primary_LUAD_with_Met
Erythroblast	Erythroblast_Normal_Lung
	Erythroblast_Subsolid_Nodule
	Erythroblast_Primary_LUAD_with_Met

RCC_refPhi

Cell Types	Cell States
Plasma	Plasma_Plasma-IgG
	Plasma_Plasma-IgA
	Plasma_Plasma-cycling
B-cell	B-cell_Switched-memory_B
	B-cell_Naïve_B.2
	B-cell_Naïve_B.1
	B-cell_Activated_B-AREG
	B-cell_Stress_B
	B-cell_Activated_B-RHOB
	B-cell_INF-response_B
	B-cell_Non-switched-memory_B
T-cell	T-cell_CD4+T_EM
	T-cell_CD4+T_Naïve/CM
	T-cell_CD4+Treg
	T-cell_CD4+T_Naïve/CM-patient-specific.1
	T-cell_CD4+T_INF-response
	T-cell_CD4+T_Act-CXCR4
	T-cell_CD4+T_Act-NR4A1
	T-cell_CD4+T_Act-CCL5
	T-cell_CD4+T_Act-CH25H
	T-cell_CD4+T_Naïve/CM-patient-specific.2
	T-cell_CD8+T_EM
	T-cell_gdT_Vd2
	T-cell_CD8+T_EMRA
	T-cell_CD8+T_Naïve/CM
	T-cell_CD8+T_Cycling-G2/M
	T-cell_MAIT
	T-cell_CD8+T_Cycling-G1/S
	T-cell_gdT_Vd1
	T-cell_CD8+T_EX-IL10
	T-cell_CD8+T_EX-CCL4L2
	T-cell_CD8+T_preEX-CXCR4
	T-cell_CD8+T_EFF.2
	T-cell_CD8+T_EFF.1
	T-cell_CD8+T_Act-XCL1/2
	T-cell_CD8+T_RM-CXCL13
	T-cell_CD8+T_preEX-PMCH
Endo	EC_CCL5+_EC
	EC_ACKR+_EC
	EC_Collagen_EC

RCC_refPhi (continued)

Cell Types	Cell States
	EC_DNASE1L3+_EC
	EC_IGF2+_EC
	EC_CRHBP+_EC
	EC_Cycling_EC
	EC_Pericyte
	EC_IGFBP3+_EC
	EC_FABP4+_EC
	EC_Lymphatic_EC
Fibro	Fibro_CYGB+ Fibro
	Fibro_MHC-II Fibro
	Fibro_CNN1+ Fibro
	Fibro_Collagen Fibro
	Fibro_MMP Fibro
	Fibro_CDH19+ Fibro
	Fibro_Stress Fibro
	Fibro_SMC-like Fibro
	Fibro_NFKBIZ+ Fibro
Myeloid	Myeloid_Non-classical Mono
	Myeloid_Classical Mono.2
	Myeloid_Classical Mono.4
	Myeloid_Classical Mono.3
	Myeloid_TR Mac.2
	Myeloid_Classical Mono.1
	Myeloid_RGS+ TAM
	Myeloid_Pro-infla. TAM
	Myeloid_MHC-II TAM
	Myeloid_TR Mac.1
	Myeloid_GPNMB+ TAM
	Myeloid_TR Mac.3
	Myeloid_SPP1+ TAM
	Myeloid_FN1+ TAM
DC	pDC_pDC
	Myeloid_pDC
	Myeloid_cDC1
	Myeloid_cDC2
Mast	Mast_Mast cell
NK	NK_NK-CX3CR1-high
	NK_cir_NK-PTGDS
	NK_NK-MTRNR2L12
	NK_ILC

RCC_refPhi (continued)

Cell Types	Cell States
	NK_Inflam NK
	NK_Activated NK
	NK_NK-IFNG-high
	NK_cir_NK-S100B
	NK_Activated KRT+ NK
	NK_NK-FXYD2
	NK_NK-SPARC
	NK_NK-CD65-bright
Malignant	RCC_PT
	RCC_MHCII
	RCC_EMT
	RCC_Cycling
	RCC_Stress
	RCC_CD
Epi	Epi_non-PT_CRYAB+ Epi
	Epi_non-PT_LoH ATL
	Epi_non-PT_LoH TAL
	Epi_non-PT_DCT cell
	Epi_non-PT_Type A intercalated cell
	Epi_non-PT_CD principal cell
	Epi_non-PT_Connecting tubule cell
	Epi_non-PT_Type B intercalated cell
	Epi_non-PT_Pelvic urothelial cell
	Epi_PT_CRYAB+ Epi
	Epi_PT_PT1/2
	Epi_PT_PT3

OV_refPhi

Cell Types	Cell States
malignant	Cycling.cancer.cell.2
	Cycling.cancer.cell.1
	fallopian tube secretory epithelial cell
	Cancer.cell.1
	Cancer.cell.2
	Cancer.cell.6
	Ciliated.cell.1
	Cancer.cell.3
	Cancer.cell.4
	Cancer.cell.5
	Ciliated.cell.2
fibroblast	fibroblast
T cell	T cell
	CD8.T.cytotoxic
	CD4.T.reg
	CD4.T.naive
	Cycling.T.NK
	CD8.T.ISG
	CD8.T.CXCL13
	CD4.T.CXCL13
	NK.CD56
	NK.cytotoxic
	ILC
endothelial cell	endothelial cell
plasma cell	plasma cell
B cell	B cell
dendritic cell	pDC
	mDC
	cDC2
	cDC1
mast cell	Mast.cell
monocyte	Cycling.M
	M1.S100A8
	M2.CXCL10
	M2.SELENOP
	M2.MARCO
	monocyte
	Clearing.M
	M2.MMP9
	M2.COL1A1

SKCM_refPhi

Cell Types	Cell States
B cells	B cells
Melanoma cells	Melanoma cells_tumor71
	Melanoma cells_tumor81
	Melanoma cells_tumor80
	Melanoma cells_tumor79
	Melanoma cells_tumor82
	Melanoma cells_tumor53
	Melanoma cells_tumor59
	Melanoma cells_tumor65
	Melanoma cells_tumor78
	Melanoma cells_tumor84
	Melanoma cells_tumor60
	Melanoma cells_tumor88
	Melanoma cells_tumor89
	Melanoma cells_tumor94
Macrophages/Monocytes	Macrophages/Monocytes
Cancer associated fibroblasts	Cancer associated fibroblasts
Dendritic cells	Dendritic cells
Endothelial cells	Endothelial cells
Natural killer cells	Natural killer cells
Tcell	CD8+ T cells
	CD4+ T cells
	regulatory T cells

Table S3: Summary of TCGA datasets used in reference evaluation

reference	TCGA Dataset	number of samples	#samples with ABSOLUTE[1] info	number of samples with CPE[2] info	#samples with ESTIMATE[3] info	#samples with IHC[4] info	#samples with LUMP[5] info
BRCA_refPhi	TCGA-BRCA	1,217	1,032	1,085	1,088	1,101	729
CRC_refPhi	TCGA-COAD/READ [6]	689	529	631	382	634	393
GBM_refPhi	TCGA-GBM	173	152	162	165	167	59
LUAD_refPhi	TCGA-LUAD	585	489	523	510	521	456
OV_refPhi	TCGA-OV	379	249	374	231	379	7
RCC_refPhi	TCGA-KIRC	607	326	533	531	535	321
SKCM_refPhi	TCGA-SKCM	472	459	467	469	470	459

1: ABSOLUTE: Somatic copy-number data based purity estimation method [5]
2: CPE: Consensus purity estimation method integrating purity estimates from ABSOLUTE, ESTIMATE, LUMP, and IHC [7]
3: ESTIMATE: Gene expression based purity estimation method [6]
4: IHC: Image analysis of haematoxylin and eosin stain slides produced by the Nationwide Children's Hospital Biospecimen Core Resource [7]
5: LUMP: DNA methylation based purity estimation method [7]
6: both colon adenocarcinoma and rectum adenocarcinoma samples are collected from TCGA for CRC_refPhi validation

References

[1] Tinyi Chu et al. Cell type and gene expression deconvolution with BayesPrism enables Bayesian integrative analysis across bulk and single-cell RNA sequencing in oncology. *Nature Cancer*, 3(4):505–517, April 2022. ISSN 2662-1347. doi: 10.1038/s43018-022-00356-3.

[2] David M. Blei, Andrew Y. Ng, and Michael I. Jordan. Latent dirichlet allocation. *Journal of machine Learning research*, 3(Jan):993–1022, 2003.

[3] Lakshmipuram S. Swapna, Michael Huang, and Yue Li. GTM-decon: guided-topic modeling of single-cell transcriptomes enables sub-cell-type and disease-subtype deconvolution of bulk transcriptomes. *Genome Biology*, 24(1):190, 2023. doi: 10.1186/s13059-023-03034-4.

[4] Tinyi Chu et al. BayesPrism tutoral data: https://github.com/danko-lab/bayesprism/tree/main/tutorial.dat. Accessed Jan 2022.

[5] Scott L. Carter et al. Absolute quantification of somatic DNA alterations in human cancer. *Nature Biotechnology*, 30(5):413–421, 2012. doi: 10.1038/nbt.2203.

[6] Kosuke Yoshihara et al. Inferring tumour purity and stromal and immune cell admixture from expression data. *Nature Communications*, 4:2612, 2013. doi: 10.1038/ncomms3612.

[7] Dvir Aran, Marina Sirota, and Atul Butte. Systematic pan-cancer analysis of tumour purity. *Nature Communications*, 6:8971, 2015. doi: 10.1038/ncomms9971.

[8] Mengying Hu and Maria Chikina. Heterogeneous pseudobulk simulation enables realistic benchmarking of cell-type deconvolution methods. *Genome Biology*, 25(1):169, 2024. ISSN 1474-760X. doi: 10.1186/s13059-024-03292-w.

[9] Ariel A. Hippen et al. Performance of computational algorithms to deconvolve heterogeneous bulk ovarian tumor tissue depends on experimental factors. *Genome Biology*, 24(1):239, 2023. doi: 10.1186/s13059-023-03077-7.

[10] Lana X. Garmire et al. Challenges and perspectives in computational deconvolution of genomics data. *Nature Methods*, 21:391–400, 2024. doi: 10.1038/s41592-023-02166-6.

[11] Assif Gavish et al. Hallmarks of transcriptional intratumour heterogeneity across a thousand tumours. *Nature*, 618:598–606, 2023. doi: 10.1038/s41586-023-06130-4.

[12] CZI Single-Cell Biology Program et al. CZ CELL×GENE Discover: A single-cell data platform for scalable exploration, analysis and modeling of aggregated data. *bioRxiv*, 2023. doi: 10.1101/2023.10.30.563174.

[13] Leyla Tarhan et al. Single cell portal: an interactive home for single-cell genomics data. *bioRxiv*, 2023. doi: 10.1101/2023.07.13.548886.

[14] Ruochen Jiang et al. Statistics or biology: the zero-inflation controversy about scRNA-seq data. *Genome Biology*, 23:31, 2022. doi: 10.1186/s13059-022-02601-5.

[15] Aaron T. L. Lun, Davis J. McCarthy, and John C. Marioni. A step-by-step workflow for low-level analysis of single-cell RNA-seq data with Bioconductor. *F1000Research*, 5:2122, 2016. doi: 10.12688/f1000research.9501.2.

[16] Jack R. Leary et al. Sub-cluster identification through semi-supervised optimization of rare-cell silhouettes (scissors) in single-cell RNA-sequencing. *Bioinformatics*, 39(8), 2023. doi: 10.1093/bioinformatics/btad449.

[17] Francisco Avila Cobos et al. Benchmarking of cell type deconvolution pipelines for transcriptomics data. *Nature Communications*, 11(1):5650, November 2020. ISSN 2041-1723. doi: 10.1038/s41467-020-19015-1.

[18] Mary J. Goldman et al. Visualizing and interpreting cancer genomics data via the Xena platform. *Nature Biotechnology*, 38(6):675–678, June 2020. ISSN 1546-1696. doi: 10.1038/s41587-020-0546-8.

[19] Antonio Colaprico et al. TCGAbiolinks: an R/Bioconductor package for integrative analysis of TCGA data. *Nucleic Acids Research*, 44(8):e71, 2016. doi: 10.1093/nar/gkv1507.

[20] Brian B Nadel et al. Systematic evaluation of transcriptomics-based deconvolution methods and references using thousands of clinical samples. *Briefings in Bioinformatics*, 22(6): bbab265, 08 2021. ISSN 1477-4054. doi: 10.1093/bib/bbab265.

[21] Bernard A. Luca et al. Atlas of clinically distinct cell states and ecosystems across human solid tumors. *Cell*, 184(21):5482–5496.e28, 2021. doi: 10.1016/j.cell.2021.09.014.

[22] Karin Pelka et al. Spatially organized multicellular immune hubs in human colorectal cancer. *Cell*, 184(18):4734–4752.e20, 2021. doi: 10.1016/j.cell.2021.08.003.

[23] The Cancer Genome Atlas Network. Comprehensive molecular portraits of human breast tumours. *Nature*, 490:61–70, 2012. doi: 10.1038/nature11412.

[24] Adriana Ivich et al. Missing cell types in single-cell references impact deconvolution of bulk data but are detectable. *bioRxiv*, 2024. doi: 10.1101/2024.04.25.590992.

[25] 10x Genomics. 3k PBMCs from a Healthy Donor: https://support.10xgenomics.com/single-cell-gene-expression/datasets/2.1.0/pbmc3k. Genomics, 2016. Available from 10x Genomics.

[26] Aleksandra Bożyk et al. Tumor microenvironment—a short review of cellular and interaction diversity. *Biology*, 11(6), 2022. ISSN 2079-7737. doi: 10.3390/biology11060929.

[27] Cristian Ruiz-Moreno et al. Harmonized single-cell landscape, intercellular crosstalk and tumor architecture of glioblastoma. *bioRxiv*, 2022. doi: 10.1101/2022.08.27.505439.

[28] Mengying Hu and Maria Chikina. InstaPrism Source Code: https://github.com/humengying0907/instaprismsourcecode.

[29] Ruoyan Li et al. Mapping single-cell transcriptomes in the intra-tumoral and associated territories of kidney cancer. *Cancer Cell*, 40(12):1583–1599.e10, 2022. doi: 10.1016/j.ccell.2022.11.001.

[30] Egor Revkov et al. PUREE: accurate pan-cancer tumor purity estimation from gene expression data. *Communications Biology*, 6:394, 2023. doi: 10.1038/s42003-023-04764-8.

[31] Valsamo Anagnostou et al. Multimodal genomic features predict outcome of immune checkpoint blockade in non-small-cell lung cancer. *Nature Cancer*, 1:99–111, 2020. doi: 10.1038/s43018-019-0008-8.

[32] Mengyuan Li et al. An algorithm to quantify intratumor heterogeneity based on alterations of gene expression profiles. *Communications Biology*, 3:505, 2020. doi: 10.1038/s42003-020-01230-7.

[33] Aaron M. Newman et al. Determining cell type abundance and expression from bulk tissues with digital cytometry. *Nature Biotechnology*, 37(7):773–782, July 2019. ISSN 1546-1696. doi: 10.1038/s41587-019-0114-2.

[34] Julien Racle et al. Simultaneous enumeration of cancer and immune cell types from bulk tumor gene expression data. *eLife*, 6:e26476, 2017. doi: 10.7554/eLife.26476.

[35] Ting Gong and Joseph D. Szustakowski. DeconRNASeq: a statistical framework for deconvolution of heterogeneous tissue samples based on mRNA-Seq data. *Bioinformatics*, 29(8): 1083–1085, 2013. doi: 10.1093/bioinformatics/btt090.

[36] Zeya Wang et al. Transcriptome deconvolution of heterogeneous tumor samples with immune infiltration. *iScience*, 9:451–460, 2018. doi: 10.1016/j.isci.2018.10.028.

[37] Sunny Z. Wu et al. A single-cell and spatially resolved atlas of human breast cancers. *Nature Genetics*, 53(9):1334–1347, September 2021. ISSN 1061-4036. doi: 10.1038/s41588-021-00911-1.

[38] Ayse Bassez et al. A single-cell map of intratumoral changes during anti-PD1 treatment of patients with breast cancer. *Nature Medicine*, 27:820–832, 2021. doi: 10.1038/s41591-021-01323-8.

[39] Junbin Qian et al. A pan-cancer blueprint of the heterogeneous tumor microenvironment revealed by single-cell profiling. *Cell Research*, 30(9):745–762, September 2020. ISSN 1748-7838. doi: 10.1038/s41422-020-0355-0.

[40] Hae-Ock Lee et al. Lineage-dependent gene expression programs influence the immune landscape of colorectal cancer. *Nature Genetics*, 52(6):594–603, June 2020. ISSN 1546-1718. doi: 10.1038/s41588-020-0636-z.

[41] Jinzhou Yuan et al. Single-cell transcriptome analysis of lineage diversity in high-grade glioma. *Genome Medicine*, 10(1):57, Jul 2018. doi: 10.1186/s13073-018-0567-9.

[42] Cyril Neftel et al. An integrative model of cellular states, plasticity, and genetics for glioblastoma. *Cell*, 178(4):835–849.e21, 2019. ISSN 0092-8674. doi: 10.1016/j.cell.2019.06.024.

[43] Xudong Xing et al. Decoding the multicellular ecosystem of lung adenocarcinoma manifested as pulmonary subsolid nodules by single-cell RNA sequencing. *Science Advances*, 7(5), January 2021. doi: 10.1126/sciadv.abd9738.

[44] Nayoung Kim et al. Single-cell RNA sequencing demonstrates the molecular and cellular reprogramming of metastatic lung adenocarcinoma. *Nature Communications*, 11:2285, 2020. doi: 10.1038/s41467-020-16164-1.

[45] Ignacio Vázquez-García et al. Ovarian cancer mutational processes drive site-specific immune evasion. *Nature*, 612(7941):778–786, 2022. doi: 10.1038/s41586-022-05496-1.

[46] Benjamin Izar et al. A single-cell landscape of high-grade serous ovarian cancer. *Nature Medicine*, 26:1271–1279, 2020. doi: 10.1038/s41591-020-0926-0.

[47] Yuping Zhang et al. Single-cell analyses of renal cell cancers reveal insights into tumor microenvironment, cell of origin, and therapy response. *Proceedings of the National Academy of Sciences of the United States of America*, 118(24):e2103240118, 2021. doi: 10.1073/pnas.2103240118.

[48] Matthew D. Young et al. Single-cell transcriptomes from human kidneys reveal the cellular identity of renal tumors. *Science*, 361:594–599, 2018. doi: 10.1126/science.aat1699.

[49] Itay Tirosh et al. Dissecting the multicellular ecosystem of metastatic melanoma by single-cell RNA-seq. *Science*, 352(6282):189–196, 2016. doi: 10.1126/science.aad0501.

[50] Livnat Jerby-Arnon et al. A cancer cell program promotes T Cell exclusion and resistance to checkpoint blockade. *Cell*, 175(4):984–997.e24, 2018. doi: 10.1016/j.cell.2018.09.006.
